# Structural anisotropy results in mechano-directional transport of proteins across nuclear pores

**DOI:** 10.1038/s41567-024-02438-8

**Published:** 2024-05-13

**Authors:** Fani Panagaki, Rafael Tapia-Rojo, Tong Zhu, Natalie Milmoe, Patricia Paracuellos, Stephanie Board, Marc Mora, Jane Walker, Elena Rostkova, Andrew Stannard, Elvira Infante, Sergi Garcia-Manyes

**Affiliations:** 1 Single Molecule Mechanobiology Laboratory, The Francis Crick Institute, London, UK; 2 Department of Physics, Randall Centre for Cell and Molecular Biophysics, Centre for the Physical Science of Life and London Centre for Nanotechnology, King’s College London, London, UK

**Keywords:** Biological physics, Single-molecule biophysics

## Abstract

The nuclear pore complex regulates nucleocytoplasmic transport by means of a tightly synchronized suite of biochemical reactions. The physicochemical properties of the translocating cargos are emerging as master regulators of their shuttling dynamics. As well as being affected by molecular weight and surface-exposed amino acids, the kinetics of the nuclear translocation of protein cargos also depend on their nanomechanical properties, yet the mechanisms underpinning the mechanoselectivity of the nuclear pore complex are unclear. Here we show that proteins with locally soft regions in the vicinity of the nuclear-localization sequence exhibit higher nuclear-import rates, and that such mechanoselectivity is specifically impaired upon knocking down nucleoporin 153, a key protein in the nuclear pore complex. This allows us to design a short, easy-to-express and chemically inert unstructured peptide tag that accelerates the nuclear-import rate of stiff protein cargos. We also show that U2OS osteosarcoma cells expressing the peptide-tagged myocardin-related transcription factor import this mechanosensitive protein to the nucleus at higher rates and display faster motility. Locally unstructured regions lower the free-energy barrier of protein translocation and might offer a control mechanism for nuclear mechanotransduction.

## Abstract (web summary)

Protein transport across the nuclear membrane is regulated by the nuclear pore complex. Experiments now show that the rates of nuclear transport rely on the presence of locally mechanically soft regions of the transported proteins.

## Main

During mechanotransduction, extracellular mechanical forces need to propagate across the cell to eventually reach the nucleus to activate force-induced transcriptional programs^1–4^. Several mechanosensitive transcriptional regulators, such as the myocardin-related transcription factor A (MRTFA)^5^, yes-associated protein 1 (YAP)^6^ and nuclear factor kappa-light-chain-enhancer of activated B cells (NF-κB)^7^, which are in the cytoplasm under basal conditions, shuttle into the nucleus upon mechanical stimulation to initiate gene expression. Studding the nuclear envelope, the nuclear pore complex (NPC)—a 100-MDa protein complex formed by ~30 nucleoporin proteins (Nups)—is the main gateway in and out of the nucleus, and it orchestrates the continuous bidirectional nucleocytoplasmic traffic of RNA and proteins^8–10^.

Compared to other narrower biologically relevant pores, such as those found in mitochondria^11^, peroxisomes^12^ or the proteolytic machinery in bacteria^13,14^, the width of the central pore of the NPC is seemingly large (∼40–70 nm)^15–18^, and yet it is incredibly permselective^19^, mainly due to an ensemble of intrinsically disordered and extremely dynamic Nups rich in phenylalanine and glycine (FG) repeats that line the inner surface of the pore and form an effective barrier to translocation^20^. In the canonical picture, small molecules (up to ∼5 nm (∼40 kDa), although there is a soft cutoff threshold^21^) can diffuse through the NPC freely, whereas larger molecules, including ostensibly large cargos^22–24^, need to bind to nuclear transport receptors (NTRs)^23,25,26^. Briefly, in the importin-dependent ‘facilitated’ transport pathway, importin-α (Kapα) binds cargos containing a nuclear localization signal (NLS) sequence and importin-β (Kapβ), forming a trimeric complex in the cytoplasm. Conversely, in the nucleus, exportin binds to proteins featuring a nuclear export signal (NES). The reversibility and directionality of the process is fuelled by the nucleocytoplasmic Ran guanosine triphosphate gradient^27^. Although recent advances in cryo-electron microscopy (cryo-EM)^18,28–31^ have provided a refined molecular picture of the structural components delineating the NPC^28^, a unified view on the molecular mechanisms underpinning the dynamics of nucleocytoplasmic transport across the NPC is still far from complete^32^.

Several enticing and complementary physicochemical models have been proposed to explain the highly sophisticated pore selectivity based on the interactions within the FG-Nups, as well as between the FG-Nups and the translocating protein complex^33,34^. From the cargo perspective, both molecular weight (larger proteins take longer to translocate, although a systematic analysis of the mass-dependency of nuclear translocation dynamics in facilitated transport is missing)^21,25^ and the chemical composition of the exposed protein amino acids (hydrophobic residues exhibit a more favourable interaction with the surrounding FG-Nups, favouring translocation) have been mainly described as the key determinants of a protein’s passage rate across the NPC^35^. Experiments investigating this have highlighted, even in facilitated transport, and in addition to the NLS/NTR system, the much unappreciated role of the properties of the carried cargo in regulating nuclear translocation.

More recently, mechanical forces have been shown to regulate the dynamics of nuclear transport at the NPC at two different and independent levels^36^. First, external mechanical perturbations directly applied to the nucleus^37^, or internally applied from the cytoplasm by the LINC (linker of nucleoskeleton and cytoskeleton) complex, were able to elastically deform the NPC^38^, thereby increasing the nuclear import of the YAP transcription factor. Independently, a second (and orthogonal) new mechanism specifically related to the properties of the translocating cargo suggests that the mechanical stability of proteins regulates their nuclear import rate^39^. Yet the molecular mechanisms by which the NPC regulates the translocation of proteins by sensing their nanomechanical stability—and the functional consequences at the cellular level—remain unknown. How protein mechanics is linked to other relevant cargo physicochemical properties (namely mass and sequence) to collectively regulate their nuclear transport remains poorly understood. Given that transport is directional and that the mechanical resistance of proteins is a localized notion^40,41^, the challenge lies in establishing how a protein’s local mechanical stability regulates its nuclear import dynamics. Unravelling the physical rules underpinning the NPC mechanoselectivity jigsaw would potentially inspire the rational design of proteins with enhanced nuclear affinity, offering new molecular strategies towards the engineering of nuclear mechanotransduction.

**Fig. 1:**
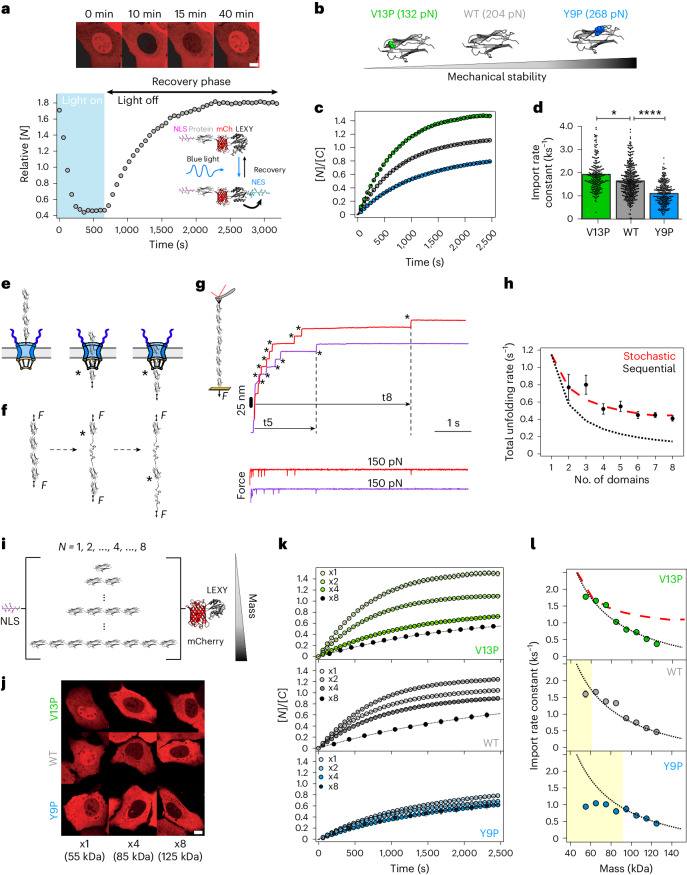
The mechanical stability of the translocating protein cargo determines its nuclear accumulation up to a mass threshold, beyond which molecular weight dominates. **a**, Optogenetics assay to measure the time course of the nucleus-to-cell localization of the NLS-X-mCherry-LEXY (here X = Ig27_WT_) probe, [*N*](*t*). Representative confocal images are shown at the top. Scale bar, 10 µm. **b**–**d**, Nuclear import kinetics of Ig27_WT_ (WT), Ig27_V13P_ (V13P) and Ig27_Y9P_ (Y9P): unfolding forces of each Ig27 variant obtained by AFM experiments^40^ (**b**); average time courses of the nucleus/cytoplasm ratio, [*N*]/[*C*](*t*), for V13P, WT and Y9P (**c**); resulting import rate constants (*k*_I_) for WT (*k*_I_ = 1.62 ± 0.05 ks^−1^, *n* = 249), V13P (*k*_I_ = 1.80 ± 0.09 ks^−1^, *n* = 161) and Y9P (*k*_I_ = 1.09 ± 0.03 ks^−1^, *n* = 279) (**d**). Significance levels (two-tailed Mann–Whitney non-parametric test): *P < 0.05; ****P < 0.0001. WT versus V13P, *P* = 0.04; WT versus Y9P, *P* = 6.60 × 10^−16^. **e**, Schematics of a polyprotein construct made of multiple identical protein domains as it sequentially translocates to the nucleus across the NPC, one domain at a time (black asterisk). **f**, By contrast, in an in vitro single polyprotein pulling experiment, in which each domain unfolds stochastically (black asterisks) with the same probability per unit time (*r*_U_d*t*), where *r*_U_ is the characteristic unfolding rate of each individual domain. **g**, Force-clamp AFM unfolding trajectory of an individual (Ig27_WT_)_8_ polyprotein stretched at 150 pN. The total unfolding time *t*_N_ depends on *N*, the number of unfolding domains (*N* = 5, purple trace; *N* = 8, red trace). **h**, Total unfolding rate *r*_N_ of an Ig27_WT_ polyprotein as a function of *N*, calculated as *r*_N_ = 1/*t*_N_. The resulting unfolding kinetics can be reproduced by a stochastic model where *r*_N_ = *r*_U_/Σ(1/*n*), with *n* = 1, …, *N* (red dashed line) and with *r*_U_ = 1.15 s^−1^. For comparison, a sequential unfolding process would exhibit a steeper dependence on *N* (black dotted line*, r*_N_ = *r*_U_/*N*). **i**, Schematics of the LEXY probe modified to include a varying number of Ig27 domains (*N* = 1–8). **j**, Representative confocal images of U2OS cells expressing the LEXY construct with protein cargos of different mechanical stabilities (V13P, WT and Y9P) and molecular weights (*N* = 1, 4 and 8 are shown), taken 30 min into the recovery phase. Scale bar, 10 µm. **k**,**l**, Average recovery curves of V13P (top), WT (middle) and Y9P (bottom) polyproteins (**k**) and the associated import rate constants (**l**) for *N* = 1–8. All points and bar plots indicate mean ± s.e.m.

We started by probing how the dynamics of nuclear entry are affected by the mechanical stability of an individual translocating protein monomer. We transiently transfected U2OS cells with a light-inducible nuclear export (LEXY) probe (AsLOV2-NES)^42^ (Fig. 1a), previously modified to contain a specific protein (X) mechanical marker, NLS-X-mCherry-LEXY^39^. As a first choice, we used X = Ig27_WT_ (titin’s 27th immunoglobulin (Ig) domain, a well-studied mechanical protein model that has been independently characterized by single-molecule atomic force microscopy (AFM)^43^) and its two mutants, Ig27_V13P_ and Ig27_Y9P_ (ref. ^40^). The introduction of a single-point (V13P, Y9P) mutation to the Ig27_WT_ results in a substantial change in its mechanical stability while keeping its fold and sequence (and hence the exposed amino acids) intact (Fig. 1b). Briefly, in the dark state, the NES of the LEXY probe is docked to the light-oxygen-voltage-sensing (LOV) domain, rendering it invisible to exportins. As a result, the construct mostly accumulates in the nucleus. Upon blue-light exposure, the NES motif is rapidly exposed, resulting in fast exclusion of the optogenetic probe from the nucleus. Switching off the light again re-docks the NES and triggers the construct to exponentially accumulate back into the nucleus (Fig. 1a). Comparing the nuclear translocation kinetics (Supplementary Fig. 1) of the optogenetic tool tagged with the three Ig27 variants in the absence of light revealed that the import (but not the export, Supplementary Fig. 2) rate of the mechanically stable Ig27_Y9P_ mutant was significantly lower than that corresponding to the Ig27_WT_ and the mechanically labile Ig27_V13P_ form (Fig. 1c,d). Altogether, these proof-of-principle experiments suggest that the rate of nuclear entry is inversely correlated with the mechanical stability of the translocating protein, in agreement with previous experiments^39^. This implies that, at least at the monomer level, the (at least partial) mechanical unfolding of Ig27 regulates its nuclear translocation rate.

To elucidate the mechanism by which structurally complex and larger protein cargos translocate into the nucleus across the NPC, we employed (homo)polyproteins made of identical repeats of the same protein monomer. It is tempting to speculate that, when entering the pore, a polyprotein made of several independent protein monomers will translocate one monomer at a time (sequential translocation) as they find the pore mouth (Fig. 1e). This scenario is met in the ClpX bacterial proteolytic machine^14,44^, where individual mechanically stable domains independently unfold as they encounter the proteolytic pore, as shown by single-molecule techniques. For such a sequential translocation scenario, the total translocation rate of the entire polyprotein (*r*_*N*_) scales with the number of domains (*N*) as ${{r}_{{\mathrm{N}}}\left(N\right)}={\frac{{r}_{\rm{U}}}{N}}$, where *r*_U_ is the translocation rate of each monomer. However, given that the NPC is seemingly deep (∼40 nm)^45^, it is also possible that several domains enter the pore at once, thus being concomitantly exposed to mechanical force. This second scenario would be closer to that experienced by individual polyproteins when stretched in vitro using single-molecule techniques^46^ (Fig. 1f). In this situation, given that the force is rapidly propagated through the entire polyprotein backbone, all domains are equally exposed to force and hence their unfolding probability at a given time is identical, regardless of their position within the polyprotein chain (stochastic unfolding)^47^. Those two distinct unfolding scenarios can be singled out according to their associated dependency of the total unfolding rate *r*_N_ with the number of domains (*N*). To experimentally evaluate the dependency of the total unfolding rate as a function of *N* in this ‘stochastic unfolding’ framework, we first studied the in vitro unfolding dynamics of an individual polyprotein formed by eight identical repeats, (Ig27_WT_)_8_, when stretched at a constant force of 150 pN by a single-molecule AFM operating in force-clamp mode (Fig. 1g). A single unfolding trajectory resembles a staircase, where each individual step corresponds to the unfolding of an individual Ig27_WT_ domain within the polyprotein chain^48^. In those experiments, the protein is typically randomly picked up from the surface by the AFM tip from different positions within the chain^49^, resulting in unfolding trajectories of (up to) the full length (*N* = 8 steps). We sorted individual trajectories with the same *N* (ensuring a long detachment time to avoid biasing the overall unfolding rate distribution^50^), and we computed, in each case, the total unfolding rate (Fig. 1h). The resulting unfolding rate follows a shallow dependency with *N*, namely ${r}_{\rm{N}}{(N\;)}={\frac{{r}_{\rm{U}}}{\mathop{\sum }\nolimits_{{\rm{n=1}}}^{N}\frac{1}{n}}}$ (ref. ^47^), compatible with a stochastic model where each domain unfolds independently (segmented red line).

**Extended Data Fig. 1:**
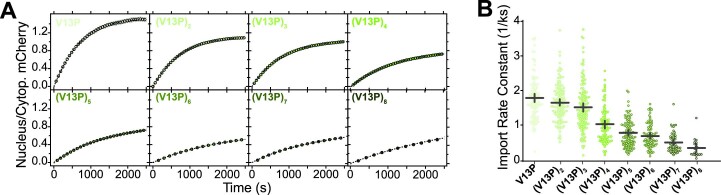
Translocation kinetics of the Ig27_V13P_ polyprotein. **(A)** Nucleus-to-cytoplasm time course localization of the construct NLS-(Ig27_V13P_)_X_-mCherry-LEXY, being X the number of Ig27_V13P_ (V13P) domains. **(B)** Import rate constants for the V13P polyproteins. Each construct was measured on at least three independent experiments, with *n* = 105 (V13P); *n* = 138 (V13P)_2_; *n* = 132 (V13P)_3_; *n* = 88 (V13P)_4_; *n* = 108 (V13P)_5_; *n* = 80 (V13P)_6_; *n* = 55 (V13P)_7_; *n* = 22 (V13P)_8_. Horizontal bars indicate the average import rate constant, and vertical bars the SEM.

**Extended Data Fig. 2:**
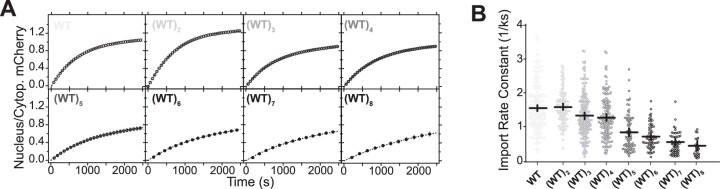
Translocation kinetics of the Ig27_WT_ polyprotein. **(A)** Nucleus-to-cytoplasm time course of the localization of a NLS-(Ig27_W**T**_)_X_-mCherry-LEXY construct, being X the number of Ig27_WT_ (WT) domains. **(B)** Import rate constants for the Ig27_WT_ polyproteins. Each construct was measured on at least three independent experiments, with *n* = 249 (WT); *n* = 115 (WT)_2_; *n* = 171 (WT)_3_; *n* = 159 (WT)_4_; *n* = 85 (WT)_5_; *n* = 68 (WT)_6_; *n* = 61 (WT)_7_; *n* = 27 (WT)_8_. Horizontal bars indicate the average import rate constant, and vertical bars the SEM.

**Extended Data Fig. 3:**
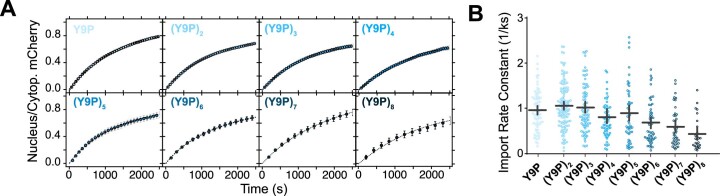
Translocation kinetics of the Ig27_Y9P_ polyprotein. **(A)** Nucleus-to-cytoplasm time course localization of the NLS-(Ig27_Y9P_)_X_-mCherry-LEXY construct, being X the number of Ig27_Y9P_ (Y9P) domains. **(B)** Import rate constants for the Y9P polyproteins. Each construct was measured on at least three independent experiments, with *n* = 78 (Y9P); *n* = 127 (Y9P)_2_; *n* = 83 (Y9P)_3_; *n* = 58 (Y9P)_4_; *n* = 50 (Y9P)_5_; *n* = 56 (Y9P)_6_; *n* = 40 (Y9P)_7_; *n* = 27 (Y9P)_8_. Horizontal bars indicate the average import rate constant, and vertical bars the SEM.

To examine the mechanism of nuclear translocation of Ig27 polyproteins in U2OS cells, we used the optogenetic approach to systematically study the rate of nuclear translocation of Ig27_WT_, Ig27_V13P_ and Ig27_Y9P_ polyproteins of increasing mass (Ig27)_N_, with 1 ≤ *N* ≤ 8 (Fig. 1i). For each polyprotein length, we measured the dynamics of nuclear accumulation (Fig. 1j) and calculated the associated nuclear import rate (Fig. 1k,l and Extended Data Figs. 1–3). In the case of Ig27_V13P_, we observed a strong mass dependency that could be nicely reproduced by an ∼(1/*N*) scaling relationship (mass law) across all probed protein lengths (1 ≤ *N* ≤ 8), reminiscent of a sequential import mechanism. An almost identical dependency was measured for the Ig27_WT_ polyproteins. However, in this case, the monomer data point (∼55 kDa) fell substantially below its expected mass law. This behaviour was exacerbated with the mechanically stronger I27_Y9P_ polyproteins, which showed a very significant slowing down of the rate of nuclear entry and an almost flat dependency for molecular weights below ∼95 kDa (*N* = 5, pentamer). These experiments suggest that, for proteins of low mechanical stability (Ig27_V13P_), the sequential model for import applies for all polyprotein lengths (molecular weights) probed. This hypothesis was further corroborated for the mechanically labile spectrin R16 polyproteins^51^, which exhibit their own mass law, and also follow the ∼(1/*N*) scaling across all polyprotein lengths (R16)_N_ (1 ≤ *N* ≤ 8) (Supplementary Fig. 3). However, as the protein’s mechanical stability increases, a deviation from the protein’s mass law emerges at low molecular weights, globally suggesting a mass threshold value—which depends on the mechanical stability of the protein— below which the rate of nuclear entry is dominated by the mechanical stability of the protein, and beyond which it is uniquely modulated by mass. A deep implication of these data is that the mechanical stability of the first protein region (or first protein domain(s)) entering the pore mouth (leading protein) seems to be the bottleneck for import dynamics.

**Fig. 2:**
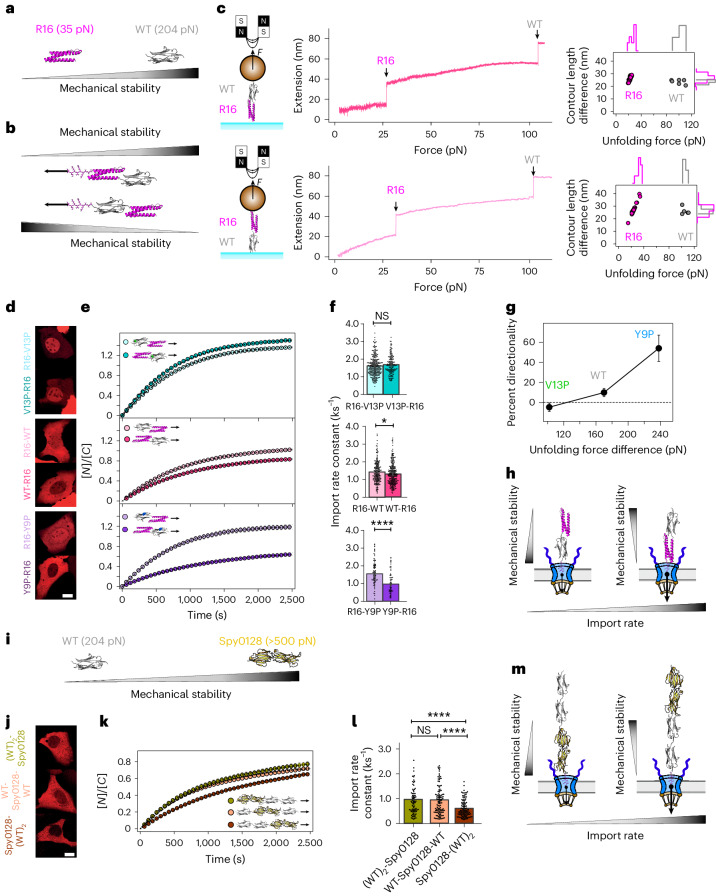
The mechano-directionality of the protein cargo determines its nuclear import kinetics across the NPC. **a**, Unfolding forces (AFM force–extension, *v* = 400 nm s^−1^)^51^ of the soft spectrin R16 domain and stiff titin Ig27_WT_ (WT). **b**, Heterodimer protein constructs alternatively placing the R16 or WT at the N-terminal position (after the NLS). **c**, Magnetic tweezers pulling experiments comparing the unfolding dynamics R16-WT (top) and WT-R16 (bottom) protein constructs. **d**–**f**, Nuclear import kinetics of heterodimers composed of the labile R16 domain, and an Ig27 variant (V13P, WT, Y9P) alternatively placed at the N- or C- terminus, to test the effect of protein mechano-directionality on the nuclear translocation kinetics. **d**, Representative confocal images of U2OS cells after 30 min into the recovery phase. Scale bar, 10 µm. **e**, Average time courses of the nucleus-to-cytoplasm localization of the respective protein construct. **f**, Import rates calculated from fits to the recovery time courses. R16-V13P (*k*_I_ = 1.64 ± 0.04 ks^−1^, *n* = 276); V13P-R16 (*k*_I_ = 1.72 ± 0.04 ks^−1^, *n* = 154); R16-WT (*k*_I_ = 1.42 ± 0.04 ks^−1^, *n* = 192); WT-R16 (*k*_I_ = 1.29 ± 0.04 ks^−1^, *n* = 260); R16-Y9P (*k*_I_ = 1.58 ± 0.07 ks^−1^, *n* = 80); Y9P-R16 (*k*_I_ = 1.02 ± 0.08 ks^−1^, *n* = 55). Significance levels for two-tailed the Mann–Whitney non-parametric test: NS*, P* > 0.05; *P < 0.05; **P < 0.01; ****P < 0.0001. R16-V13P versus V13P-R16, *P* = 0.06; R16-WT versus WT-R16, *P* = 0.02; R16-Y9P versus Y9P-R16, *P* = 1.67 × 10^−6^. **g**, Relative directionality (calculated as the ratio between the import rates of the constructs with N-terminal R16 and C-terminal R16) compared to the difference in mechanical stability between each Ig27 variant and R16. **h**, Schematics highlighting the sensitivity of the NPC to the mechano-directionality of the translocating cargo. **i**, Unfolding forces (AFM force–extension, *v* = 400 nm s^−1^)^55^ measured for the titin Ig27_WT_ (WT) and the pilin Spy0128 domain. Spy0128 is mechanically ultrastable; hence, WT plays in this case the role of the mechanically labile protein. **j**–**l**, Nuclear import kinetics of heteropolyproteins composed of two WT and one Spy0128 protein dimer. **j**, Representative confocal images of U2OS cells 30 min into the recovery phase. Scale bar, 10 µm. **k**,**l**, Average time courses of nucleus-to-cytoplasm localization (**k**) and associated import rates (**l**). (WT)_2_-Spy0128 (*k*_I_ = 0.89 ± 0.07 ks^−1^, *n* = 70); WT-Spy01_2_8-WT (*k*_I_ = 0.99 ± 0.07 ks^−1^, *n* = 118); Spy0128-(WT)_2_ (*k*_I_ = 0.56 ± 0.03 ks^−1^, *n* = 106). Significance levels for two-tailed Mann–Whitney non-parametric test: NS*, P* > 0.05; ****P < 0.0001. (WT)_2_-Spy0128 versus WT-Spy0128-WT, *P* = 0.80; WT-Spy0128-WT versus Spy0128-(WT)_2_, *P* = 5.70 × 10^−5^; (WT)_2_-Spy0128 versus Spy0128-(WT)_2_, *P* = 4.45 × 10^−5^. **m**, Schematics of the dynamics of nuclear translocation of Spy0128-(WT)_2_ versus (WT)_2_-Spy0128, highlighting the sensitivity of the NPC to the mechano-directionality of the shuttling protein cargo. All points and bar plots indicate mean ± s.e.m.

This naturally led us to systematically examine the role of the leading protein (in our case, the protein next to the NLS sequence in the LEXY optogenetic construct) in determining the overall protein import rate. We started by comparing the nuclear shuttling dynamics of a simple heterodimer composed of two domains with markedly different mechanical stabilities, R16-Ig27_WT_ (Fig. 2a), and compared this to that of its specular image, Ig27_WT_-R16 (Fig. 2b). From the mechanical perspective, both proteins were indistinguishable when pulled by single-molecule magnetic tweezers (ideally suited to measure proteins with low mechanical stability^52,53^) (Fig. 2c). In both cases, the force-ramp unfolding trajectories showed first the unfolding of the mechanically labile R16 domain at low forces (∼30 pN, 21 nm step), followed by unfolding of the mechanically stable Ig27_WT_ at much higher forces (∼100 pN, 25 nm step). This was expected, because the mechanical unfolding of polyproteins composed of mechanically distinct monomers follows a hierarchy in their mechanical stability, implying that soft domains unfold first, irrespective of their sequence position within the polyprotein chain^46,54^. In addition to being mechanically analogous, both R16-Ig27_WT_ and Ig27_WT_-R16 proteins have the same overall structure (and consequently the same exposed amino acids^35^) and exactly the same mass, so one should a priori expect them to exhibit the same nuclear import rate.

However, we observed, using the nuclear optogenetic approach (Fig. 2d–f), that the construct starting with the mechanically soft R16 domain exhibited a significantly faster import rate and higher nuclear accumulation than the one starting with the mechanically rigid Ig27_WT_ domain. This was the case across the three Ig27 variants. Interestingly, for the constructs containing the mechanically rigid Ig27_Y9P_ mutant protein, the increase in nuclear accumulation of the R16-Ig27_Y9P_ as opposed to the reverse Ig27_Y9P_-R16 construct was particularly enhanced (Fig. 2g,h). The same result was independently observed in HeLa and NIH 3T3 cell lines (Supplementary Fig. 4).

**Extended Data Fig. 4:**
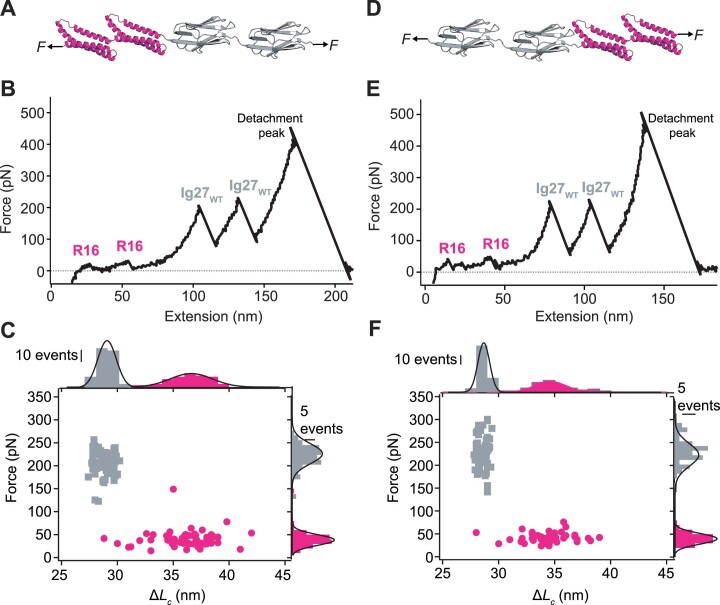
The (R16)_2_-(Ig27_WT_)_2_ and (Ig27_WT_)_2_-(R16)_2_ specular polyprotein constructs are mechanically equivalent. **(A)** Schematics of the (R16)_2_-(Ig27_WT_)_2_ polyprotein construct under force. **(B)** Representative AFM force-extension unfolding trajectory of the (R16)_2_-(Ig27_WT_)_2_ construct. The protein domains unfold following their mechanical hierarchy. The two first unfolding events, fingerprinted by an unfolding force (*F*_U_) of ∼35 pN and an increase in contour length (*ΔL*_C_) of ∼37 nm, correspond to the unfolding of the two mechanically labile R16 domains. The two subsequent events, hallmarked by *F*_U_∼220 pN with *ΔL*_C_∼27 nm, correspond to the unfolding of the stiffer Ig27_WT_. **(C)** Scatter plot of unfolding force (*F*_U_) versus increase in contour length (*ΔL*_C_) for all unfolding events detected on the AFM force–extension trajectories for the (R16)_2_-(Ig27_WT_)_2_ construct. Two clusters of events can be clearly identified by their unique *F*_U_ and *ΔL*_C_ signature, a first one with *F*_U_ = 33 ± 12 pN and *ΔL*_C_ = 37 ± 4 nm corresponding to the unfolding of the R16 domains (magenta), and a second one with *F*_U_ = 204 ± 20 pN and *ΔL*_C_ = 27 ± 2 nm corresponding to the Ig27_WT_ domains. Data collected from *n* = 61 (R16), *n* = 78 (Ig27_WT_), unfolding events from 41 individual force-extension unfolding trajectories. **(D-F)** Schematics, force–extension trace, and *F*_U_ versus *ΔL*_C_ scatter plot for the (Ig27_W**T**_)_2_-(R16)_2_ construct, showing a first cluster of events characterized by *F*_U_ = 35 ± 12 pN, and *ΔL*_C_ = 35 ± 3 nm (R16, magenta) and a second one with *F*_*u*_ = 222 ± 25 pN and *ΔL*_C_ = 28 ± 2 nm (Ig27_W**T**_, grey). When pulled in a single-molecule AFM, both specular constructs are mechanically indistinguishable. Data collected from *n* = 48 (R16), *n* = 68 (WT), unfolding events from 43 force-extension individual trajectories measured at 400 nm/s.

**Extended Data Fig. 5:**
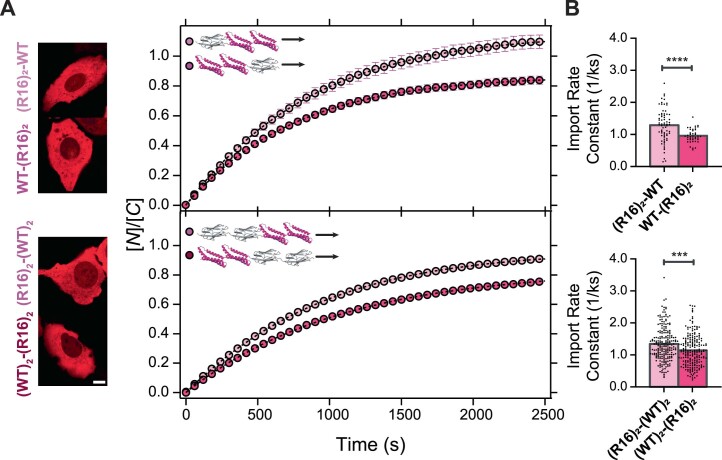
Polyproteins exhibiting a low mechanical stability domain at the N-terminus wi translocate faster to the nucleus. (**A**) Left: Representative confocal images of U2OS cells 30 min in the recovery phase. Scale bar 10 µm. Right: Average time courses of the relative nucleus-to-cytoplasm localization of (upper) (R16)_2_-WT and WT-(R16)_2_, and (lower) (R16)_2_-(WT)_2_ and (WT)_2_-(R16)_2_ protein constructs. **(B)** Protein import rate constants (mean ± SEM) calculated from the accumulation curves. (R16)_2_-WT (*k*_I_ = 1.29 ± 0.06 ks^−1^, *n* = 69); WT-(R16)_2_ (*k*_I_ = 1.02 ± 0.07 ks^−1^, *n* = 42); (R16)_2_-(WT)_2_ (*k*_I_ = 1.34 ± 0.03 ks^−1^, *n* = 217); (WT)_2_-(R16)_2_, (*k*_I_ = 1.14 ± 0.03 ks^−1^, *n* = 208). Significance levels for two-tailed Mann-Whitney non-parametric test. *** *P* ≤ 0.001, **** *P* ≤ 0.0001 (R16)_2_-(WT)_2_ vs. (WT)_2_-(R16)_2_, *P* = 9.92 × 10^−3^; (R16)_2_-(WT)_2_ vs. (WT)_2_-(R16)_2_, *P* = 1.06 × 10^−4^. All points and bar plots indicate mean ± SEM.

These experiments suggest that the NPC senses the mechano-directionality of the translocating protein cargo, in the sense that those proteins exhibiting a mechanically soft region next to the NLS tag exhibit a faster and enhanced accumulation, and that this effect is more obvious for those constructs where the gap in the mechanical stability between the ‘soft’ and ‘stiff’ regions is larger. This behaviour was maintained for larger protein constructs such as (R16)_2_-(Ig27_WT_) versus (Ig27_WT_)-(R16)_2_, (R16)_2_-(Ig27_WT_)_2_ versus (Ig27_WT_)_2_-(R16)_2_, and (Ig27_WT_)_2_-(R16)_2_ versus (Ig27_WT_-R16)_2_, which, although they have the same mechanical stability (as measured with single-molecule AFM; Extended Data Fig. 4), they display markedly different nuclear import kinetics (Extended Data Fig. 5 and Supplementary Figs. 5 and 6) when the mechanically soft domain (R16) is next to the NLS in the LEXY probe. To explore the range of cargo mechanical stabilities for which the NPC exhibits mechano-directionality, we tested the effect of sequence (and mechanical) asymmetry on the dynamics of nuclear entry for the constructs (Spy0128)-(Ig27_WT_)_2_, (Ig27_WT_)_2_-Spy0128 and Ig27_WT_-Spy0128-Ig27_WT_, where Spy0128 is a two-domain pilin protein of *Streptococcus pyogenes* that exhibits ultra-high mechanical stability due to a covalent isopeptide bond close to the protein termini that renders the protein inextensible (Fig. 2i)^55^. Note that, in these constructs, the Ig27_WT_ now acts as the soft protein, and the Spy0128 protein is the stiffer one. As before, despite their mass and overall structural identity, the (Ig27_WT_)_2_-Spy0128 construct exhibits a faster nuclear rate import than Spy0128-(Ig27_WT_)_2_ (Fig. 2j–l). Interestingly, the Ig27_WT_-Spy0128-Ig27_WT_ construct (with only one Ig27_WT_ leading) displays a nuclear import rate very close to (Ig27_WT_)_2_-Spy0128 and much faster than Spy0128-(Ig27_WT_)_2_, again suggesting that although folded, inextensible proteins such as Spy0128 can translocate across the NPC, softer leading proteins increase their nuclear import kinetics, being the main determinant of the overall import kinetics of the whole protein construct (Fig. 2m). This demonstrates that the NPC mechano-directionality is manifested for a wide range of protein mechanical stabilities.

**Fig. 3:**
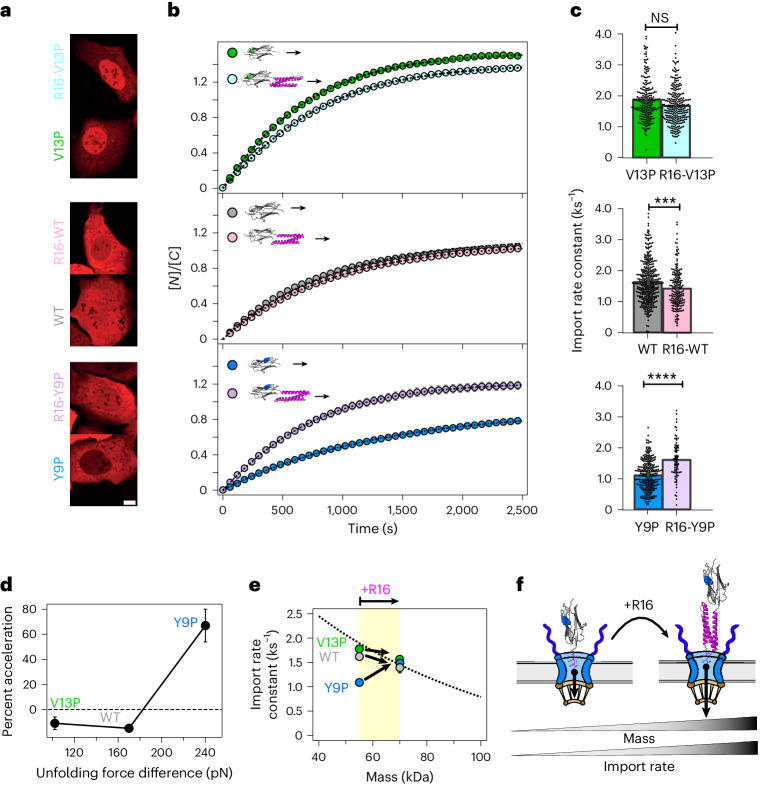
Mechanically soft leading proteins increase nuclear import. **a**–**c**, Nuclear import kinetics of R16-(V13P/WT/Y9P) compared to their Ig27 respective monomer variant: representative confocal images of U2OS cells after 30 min into the recovery phase (scale bar, 10 µm) (**a**); average time courses of the relative nucleus-to-cytoplasm localization of each protein construct (**b**); related import rates for V13P (*k*_I_ = 1.80 ± 0.09 ks^−1^, *n* = 161), R16-V13P (*k*_I_ = 1.64 ± 0.04 ks^−1^, *n* = 276); WT (*k*_I_ = 1.62 ± 0.05 ks^−1^, *n* = 249), R16-WT (*k*_I_ = 1.42 ± 0.04 ks^−1^, *n* = 192); Y9P (*k*_I_ = 1.09 ± 0.03 ks^−1^, *n* = 279), R16-Y9P (*k*_I_ = 1.58 ± 0.07 ks^−1^, *n* = 80) (**c**). Significance levels for two-tailed Mann–Whitney non-parametric test: NS, *P* > 0.05; **P < 0.001; ****P < 0.0001. V13P versus R16-V13P, *P* = 0.10; WT versus R16-WT, *P* = 3.84 × 10^−3^; Y9P versus R16-Y9P, *P* = 3.70 × 10^−10^. **d**, Relative acceleration resulting from the addition of an N-terminal R16 domain as a function of the difference in mechanical stability between R16 and the Ig27 variant (V13P, WT or Y9P). **e**, Import kinetics of the V13P, WT and Y9P monomers (55 kDa) versus the construct with an N-terminal R16 domain (70 kDa). In all cases, the import kinetics are brought up to the rate set by their respective mass law. In the case of mechanically stiff proteins (Y9P), this results in a net acceleration of the import kinetics relative to the monomer alone. **f**, Schematics of the dynamics of nuclear translocation of a Y9P monomer (left) versus the R16-Y9P construct (right). Adding the soft R16 domain next to the NLS accelerates the nuclear passage of mechanically stiff cargos, despite increasing their mass. All points and bar plots indicate mean ± s.e.m.

In light of these findings, we wondered whether the addition of a soft protein region in front of a mechanically rigid one could be exploited as a strategy to accelerate the translocation of mechanically resilient proteins inside the cell nucleus. We hence compared (Fig. 3a–c) the rate of nuclear entry of each Ig27 mutant (Ig27_V13P_, Ig27_WT_ and Ig27_Y9P_) with the related construct resulting from the addition of the mechanically labile R16 protein in front (that is, R16-Ig27_V13P_, R16-Ig27_WT_ and R16-Ig27_Y9P_). Consequently, in all constructs, the mass of the cargo was substantially increased (more than doubled) with respect to that of the Ig27 monomer alone. For both Ig27_V13P_ and Ig27_WT_ proteins, addition of the R16 did not significantly slow down their nuclear translocation dynamics in spite of their mass increase. Remarkably, in the case of the Ig27_Y9P_, addition of the R16 protein not only did not slow down its nuclear import rate, it significantly accelerated it (Fig. 3d). In all three cases, addition of the mechanically soft R16 domain compensated the mechanical stability penalty, bringing the import rate of the three constructs to their expected position in their respective mass-law relationship (Fig. 3e). In summary, addition of the mechanically soft R16 in front of mechanically stiffer monomers has a net acceleration effect on those mechanically stiff proteins (Fig. 3f).

**Fig. 4:**
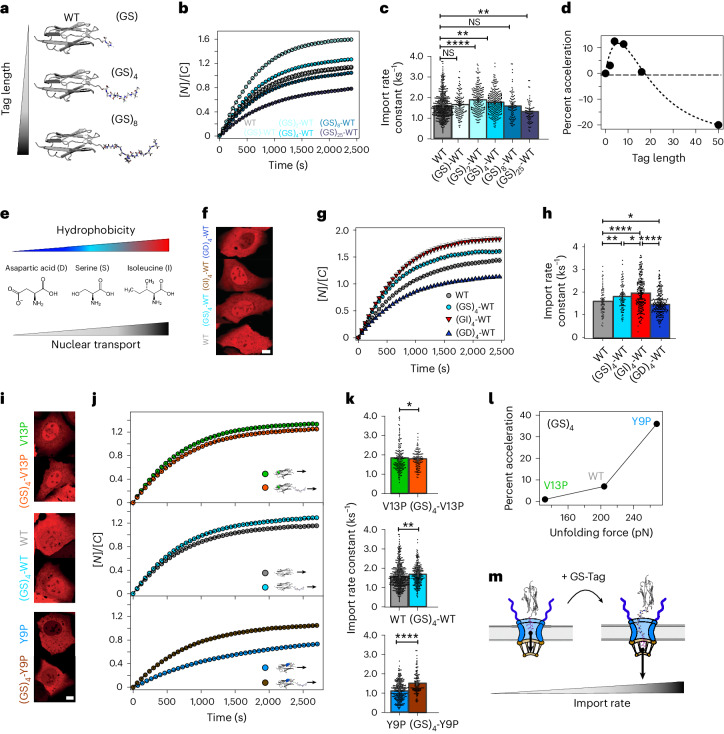
Molecular design of unstructured, mechanically labile tags to accelerate nuclear import kinetics. **a**, Addition of an N-terminal unstructured and flexible glycine–serine peptide tag to accelerate nuclear import. **b**,**c**, Average time courses of the nucleus-to-cytoplasm mCherry localization of LEXY constructs containing a (GS)_X_-WT protein cargo, (*X* = 1, 2, 4, 8, 25) (**b**) and their associated import rates (**c**): WT (*k*_I_ = 1.64 ± 0.05 ks^−1^*, n* = 249), (GS)-WT (*k*_I_ = 1.65 ± 0.07 ks^−1^, *n* = 131), (GS)_2_-WT (*k*_I_ = 1.88 ± 0.04 ks^−1^, *n* = 131), (GS)_4_-WT (*k*_I_ = 1.82 ± 0.03 ks^−1^, *n* = 357), (GS)_8_-WT (*k*_I_ = 1.61 ± 0.07 ks^−1^, *n* = 89), (GS)_25_-WT (*k*_I_ = 1.29 ± 0.06 ks^−1^, *n* = 70). Significance levels for the two-tailed Mann–Whitney non-parametric test: NS, *P* > 0.05; **P < 0.001; ****P < 0.0001. WT versus (GS)-WT, *P* = 0.44; WT versus (GS)_2_-WT, *P* = 1.67 × 10^−10^; WT versus (GS)_4_-WT, *P* = 2.46 × 10^−3^; WT versus (GS)_8_-WT, *P* = 0.94; WT versus (GS)25-WT, *P* = 1.91 × 10^−3^. **d**, Relative acceleration of the WT monomer as a function of the GS-tag length (number of amino acids). The WT is optimally accelerated when adding a flexible peptide of four to eight amino acids. **e**, The chemical properties of translocating amino acids regulate transport kinetics through the NPC, whereas charged residues like aspartic acid (D) impede NPC passage, and highly hydrophobic ones like isoleucine (I) facilitate nuclear import. **f**–**h**, Nuclear import kinetics of WT, (GS)_4_-(WT), (GI)_4_-(WT) and (GD)_4_-(WT). **f**, Representative confocal images of U2OS cells after 30 min into the recovery phase. Scale bar, 10 µm. **g**, Average time courses of the relative nucleus-to-cytoplasm localization of the respective protein construct. **h**, Associated import rates corresponding to WT (*k*_I_ = 1.60 + −0.06 ks^−1^, *n* = 80), (GS)_4_-WT (*k*_I_ = 1.82 ± 0.07 ks^−1^, *n* = 357), (GI)_4_-WT (*k*_I_ = 1.97 ± 0.06 ks^−1^, *n* = 87), (GD)_4_-WT (*k*_I_ = 1.45 ± 0.04 ks^−1^, *n* = 98). Significance levels for the two-tailed Mann–Whitney non-parametric test: *P < 0.05; **P < 0.001; ****P < 0.0001. WT versus (GS)_4_-WT, *P* = 2.46 × 10^−3^; WT versus (GI)_4_-WT, *P* = 1.11 × 10^−6^; WT versus (GD)_4_-WT, *P* = 0.02; (GS)_4_-WT versus (GI)_4_-WT, *P* = 0.04; (GI)_4_-WT versus (GD)_4_-WT, *P* = 2.74 × 10^−11^. **i**–**k**, Nuclear import kinetics of (GS)_4_-(V13P/WT/Y9P) compared to the respective monomers. **i**, Representative confocal images of U2OS cells after 30 min into the recovery phase. Scale bar, 10 µm. **j**, Time courses of the relative nucleus-to-cytoplasm localization of the respective protein construct. **k**, Related import rates: (GS)_4_-V13P (*k*_I_ = 1.90 ± 0.05 ks^−1^, *n* = 127), V13P (*k*_I_ = 1.87 ± 0.08 ks^−1^, *n* = 170); (GS)_4_-WT (*k*_I_ = 1.82 ± 0.07 ks^−1^, *n* = 357), WT (*k*_I_ = 1.62 ± 0.05 ks^−1^, *n* = 249); (GS)_4_-Y9P (*k*_I_ = 1.48 ± 0.05 ks^−1^, *n* = 132), Y9P (*k*_I_ = 1.09 ± 0.03 ks^−1^, *n* = 279). Significance levels for the two-tailed Mann–Whitney non-parametric test: *P < 0.05; **P < 0.01; ****P < 0.0001. V13P versus (GS)_4_-V13P, *P* = 0.02; WT versus (GS)_4_-WT, *P* = 2.46 × 10^−3^; Y9P versus (GS)_4_-Y9P, *P* = 2.98 × 10. The mechanically labile (GS)_4_ tag accelerates the nuclear import of the Ig27 variants. **l**, Relative acceleration of the (GS)_4_-tagged Ig27 variant as a function of its mechanical stability. **m**, Schematic showing that the addition of a mechanically labile peptide tag next to the NLS substantially increases the kinetics of the nuclear import of protein cargos across the NPC. Significance levels for the Mann–Whitney non-parametric test: NS, *P* > 0.05; *P < 0.05; ***P < 0.001; ****P < 0.0001. All points and bar plots indicate mean ± s.e.m.

Given the markedly low mechanical stability of the R16 monomer, we then conjectured that the addition of shorter polypeptides with similarly low mechanical stability (mechanically labile tags) could potentially play the same role as R16, with the advantages of featuring a much lower molecular weight and, most importantly, being easy to introduce to essentially any target protein^56,57^. As a first candidate, we chose the GS-based tag given that it is classically used in protein engineering to create flexible loops due to its low propensity to form residual structures and that its chemical composition (glycines and serines) is not expected to substantially enhance or delay nuclear import^35^. We introduced GS-tags of different lengths, ranging from (GS) to (GS)_25_, in the N-terminus of the Ig27_WT_ protein (Fig. 4a), and compared the nuclear accumulation (Fig. 4b) and associated nuclear import rate (Fig. 4c) of each resulting construct to that of the Ig27_WT_ monomer. We first found that introducing the shortest (GS) tag did not have a significant effect, whereas the longer (GS)_25_ significantly slowed the rate of nuclear translocation, probably due to its increased mass. By contrast, we observed that those constructs composed of GS sequences of intermediate lengths—especially (GS)_2_ and (GS)_4_—were able to significantly accelerate the nuclear translocation of Ig27_WT_ (Fig. 4d).

We then explored how the chemical properties of the mechanically labile tag could further regulate the nuclear import rate of the protein cargo. We replaced the chemically inert serine (S) in the (GS)_4_ tag with the negatively charged aspartic acid (D) and the highly hydrophobic isoleucine (I), which, according to recent literature^35^, should drastically delay or enhance, respectively, nuclear import across the NPC (Fig. 4e). We compared the import kinetics of the resulting (GD)_4_-Ig27_WT_ and (GI)_4_-Ig27_WT_ proteins and found that adding the (GD)_4_ tag significantly slows down (−12%) the kinetics of nuclear entry (and nuclear accumulation) of the Ig27_WT_, whereas addition of the (GI)_4_ tag significantly increases (+20%) the Ig27_WT_’s nuclear import rate, being also ~8% faster than the (GS)_4_-tagged form (Fig. 4f–h). These experiments conclude that, at least for the specific protein cargos measured in our optogenetic assay, the ‘mechanical’ and ‘chemical’ contributions to the nuclear import rate—and also the mass contribution (Fig. 1l)—are comparable in magnitude, and can be conveniently combined to finely tune the rate of nuclear import.

**Extended Data Fig. 6:**
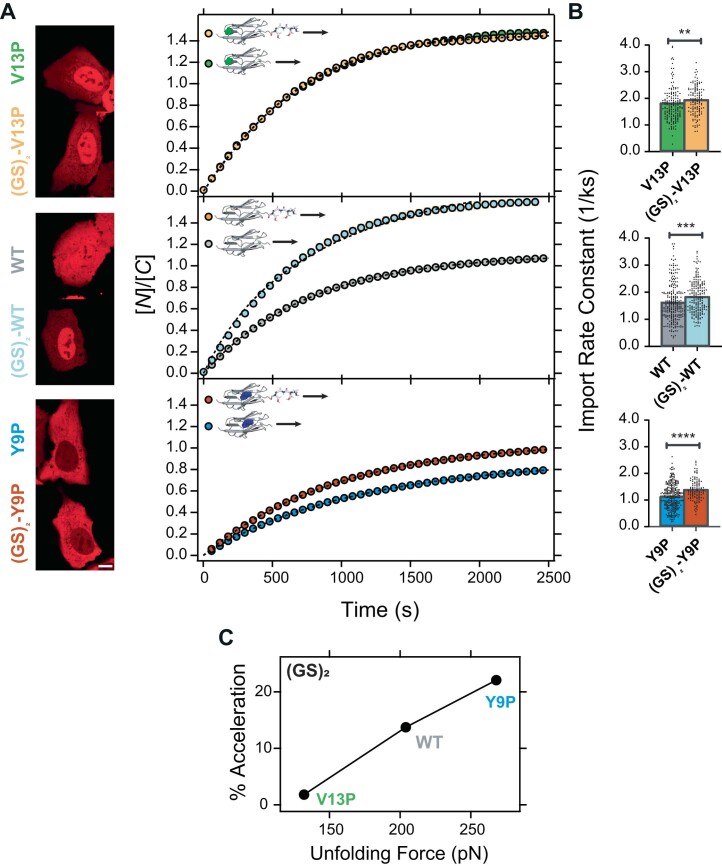
The addition of a (GS)_2_ tag accelerates nuclear import triggered by the addition of four (GS-amino acid-tag depends on the mechanical stability of the protein cargo. **(A)** Left: Representative confocal images of U2OS cells 30 min in the recovery phase. Scale bar 10 µm. Right: Average time courses of the relative nucleus-to-cytoplasm localization of (upper) V13P and (GS)_2_-V13P; (middle) WT and (GS)_2_-WT; (lower) Y9P and (GS)_2_-Y9P. **(B)** Protein import rate constants (mean ± SEM) calculated from the accumulation curves. V13P (*k*_I_ = 1.80 ± 0.09 ks^−1^, *n* = 160); (GS)_2_-V13P (*k*_*I*_ = 1.90 ± 0.05 ks^−1^, *n* = 128); WT (*k*_I_ = 1.62 ± 0.05 ks^−1^, *n* = 249); (GS)_2_-WT (*k*_I_ = 1.83 ± 0.04 ks^−1^, *n* = 196); Y9P (*k*_I_ = 1.09 ± 0.03 ks^−1^, *n* = 278); (GS)_2_-Y9P (*k*_I_ = 1.34 ± 0.04 ks^−1^, *n* = 109). Significance levels for two-tailed Mann-Whitney non-parametric test **P ≤ 0.001, ***P ≤ 0.0001, ****P ≤ 0.0001. V13P vs. (GS)_2_-V13P, *P* = 8.98 × 10^−3^; WT vs. (GS)_2_-WT, *P* = 1.17 × 10^−4^; Y9P vs. (GS)_2_-Y9P, *P* = 4.34 × 10^−5^. **(C)** Plot of the percentage acceleration induced by the (GS)_2_ addition against the unfolding force of the Ig27 variants measured in AFM force-extension experiments (*v* = 400 nm/s). All points and bar plots indicate mean ± SEM.

**Extended Data Fig. 7:**
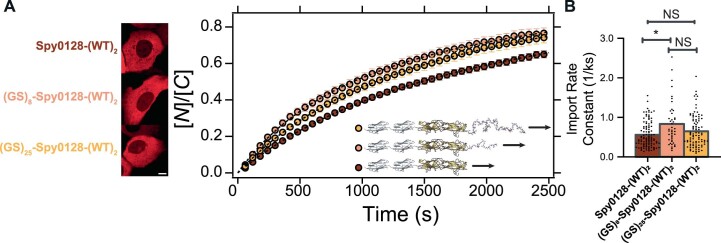
Addition of an unstructured GS-peptide tag accelerates the rate of nuclear shuttling of cargos of large mass and extremely high mechanical stability. **(A)** Left: Representative confocal images of U2OS cells 30 min in the recovery phase. Scale bar 10 µm. Right: Average time courses of the relative nucleus-to-cytoplasm localization of Spy0128-(WT)_2_, (GS)_8_-Spy0128-(WT)_2_ and (GS)_25_-Spy0128-(WT)_2_. The (GS)-tag accelerates the nuclear import kinetics of large protein cargos with extreme mechanical stability. **(B)** Protein import rate constants (mean ± SEM) calculated from the accumulation curves. Spy0128-(WT)_2_ (*k*_I_ = 0.56 ± 0.03 ks^−1^, *n* = 104); (GS)_8_-Spy0128-(WT)_2_ (*k*_I_ = 0.83 ± 0.08 ks^−1^, *n* = 43); (GS)_25_-Spy0128-(WT)_2_ (*k*_I_ = 0.65 ± 0.04 ks^−1^, *n* = 92). Significance levels for two-tailed Mann-Whitney non-parametric test NS *P* > 0.05, *P ≤ 0.05. Spy0128-(WT)_2_ vs. (GS)_8_-Spy0128-(WT)_2_, *P* = 0.01; (GS)_8_-Spy0128-(WT)_2_vs. (GS)_50_-Spy0128-(WT)_2_, *P* = 0.07; Spy0128-(WT)_2_ vs. (GS)_50_-Spy0128-(WT)_2_, *P* = 0.21. All points and bar plots indicate mean ± SEM.

We next compared the acceleration effect of the chemically inert (GS)_2_ (Extended Data Fig. 6) and (GS)_4_ in the three Ig27 variants (Fig. 4i–k), and, as before, the acceleration was substantially exacerbated for the mechanically rigid Ig27_Y9P_ mutant (Fig. 4l). It is noteworthy that the addition of GS-tags in larger (and hence slower) constructs formed by the mechanically rigid Spy0128 protein also increased their nuclear import dynamics (Extended Data Fig. 7). Consequently, these experiments demonstrate that engineering short, unstructured and floppy GS-tags is a successful molecular strategy to accelerate the nuclear import of proteins (Fig. 4m). Similar results were obtained in HeLa cells (Supplementary Fig. 7). Altogether, the global picture that emerges suggests that low-mass, mechanically soft proteins follow the kinetics dictated by their mass law, whereas mechanically rigid ones exhibit notably slowed kinetics. Such a mechanical penalty can be reversed or bypassed by the addition of a mechanically soft protein (such as the R16) at the leading end, or by adding a mechanically labile, chemically inert^35^ and conformationally disordered tag.

**Fig. 5:**
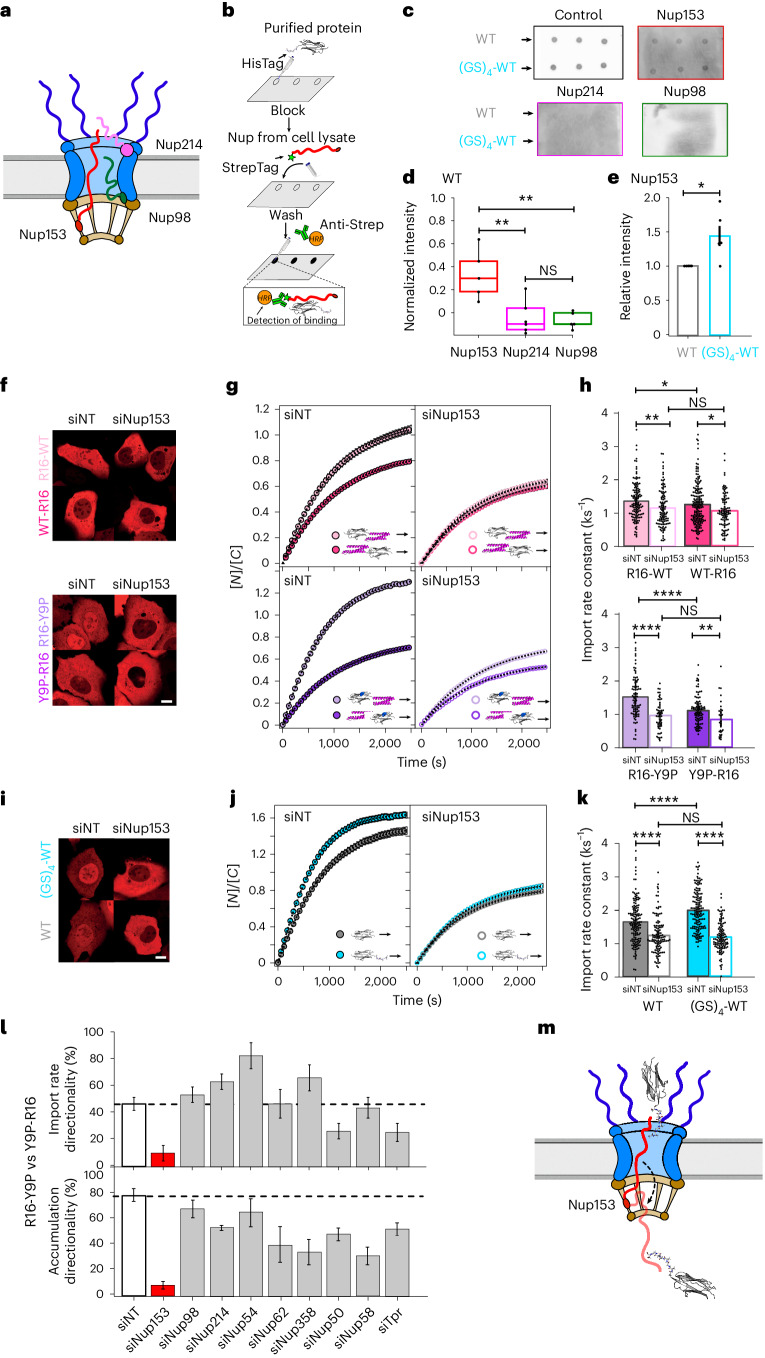
Nup153 regulates NPC sensitivity to the mechanical directionality of the translocating protein cargo. **a**, Schematics of the NPC, showing the approximate location of Nup153, Nup98 and Nup214. **b**, Schematics of the dot blot assay. **c**, Dot blot membranes to assess protein binding to Nup153, Nup98 and Nup214, including a control (no Nup present). **d**, Dot blot intensity for each of the Nups interacting with Ig27_WT_, normalized by the control experiment. Each data point corresponds to the average intensity of three dots per membrane (*N* = 5 independent dot blot experiments). Significance levels for two-tailed *t*-test: NS, *P* > 0.05; **P < 0.01; Nup153 versus Nup214, *P* = 6.94 × 10^−3^; Nup214 versus Nup98, *P* = 0.76; Nup153 versus Nup98, *P* = 1.42 × 10^−3^. Boxes span from the first to the third quartile, and the horizontal line indicates the median. **e**, Dot blot intensity for Nup153 binding to either Ig27_WT_ or (GS)_4_-Ig27_WT_. Significance levels fo the two-tailed paired *t*-test: *P < 0.05. WT versus (GS)_4_-WT, *P* = 0.04. **f**, Representative confocal images of U2OS cells 30 min into the recovery phase. Scale bar, 10 µm. **g**, Time courses of the relative nucleus-to-cytoplasm localization of R16-WT versus WT-R16 (top) and R16-Y9P versus Y9P-R16 (bottom) under normal conditions (siNT) and Nup153 knockdown (siNup153). **h**, Associated import rates: R16-WT (siNT) (*k*_I_ = 1.35 ± 0.05 ks^−1^, *n* = 130), R16-WT (siNup153) (*k*_I_ = 1.15 ± 0.05 ks^−1^, *n* = 108); WT-R16 (siNT) (*k*_I_ = 1.20 ± 0.05 ks^−1^, *n* = 179), WT-R16 (siNup153) (*k*_I_ = 1.09 ± 0.05 ks^−1^, *n* = 98); R16-Y9P (siNT) (*k*_I_ = 1.51 ± 0^.^06 ks^−1^, *n* = 92), R16-Y9P (siNup153) (*k*_I_ = 0.93 ± 0.06 ks^−1^, *n* = 57); Y9P-R16 (siNT) (*k*_I_ = 1.07 ± 0.04 ks^−1^, *n* = 100), Y9P-R16 (siNup153) (*k*_I_ = 0.83 ± 0.09 ks^−1^, *n* = 33). Significance levels for the two-tailed Mann–Whitney non-parametric test: NS, *P* > 0.05; *P < 0.05; **P < 0.01; ****P < 0.0001; siNT R16-WT versus siNup153 R16-WT, *P* = 2.14 × 10^−3^; siNT WT-R16 versus siNup153 WT-R16, *P* = 0.02; siNT R16-WT versus siNT WT-R16, *P* = 0.03; siNup153 R16-WT versus siNUP153 WT-R16, *P* = 0.25. siNT R16-Y9P versus siNup153 R16-Y9P, *P* = 5.70 × 10^−9^; siNT Y9P-R16 versus siNup153 Y9P-R16, *P* = 2.50 × 10^−3^; siNT R16-Y9P versus siNT Y9P-R16, *P* = 1.26 × 10^−7^; siNup153 R16-Y9P versus siNUP153 Y9P-R16, *P* = 0.17. **i**, Representative confocal image of U2OS cells. Scale bar, 10 µm. **j**, Time courses of the relative nucleus-to-cytoplasm localization of WT and (GS)_4_-WT under normal conditions (siNT) and Nup153 knockdown (siNup153). **k**, Associated import rates; WT (siNT) (*k*_I_ = 1.61 ± 0.05 ks^−1^, *n* = 147), WT (siNup153) (*k*_I_ = 1.22 ± 0.05 ks^−1^, *n* = 118); (GS)_4_-WT (siNT) (*k*_I_ = 2.02 ± 0.05 ks^−1^, *n* = 169), (GS)_4_-WT (siNup153) (*k*_I_ = 1.28 ± 0.04 ks^−1^, *n* = 122). Significance levels for the two-tailed Mann–Whitney non-parametric test: NS, *P* > 0.05; ****P < 0.0001; siNT WT versus siNup153 WT, *P* = 7.74 × 10^−7^; siNT (GS)_4_-WT versus siNUP153 (GS)_4_-WT, *P* = 2.50 × 10^−28^; siNT WT versus siNT (GS)_4_-WT, *P* = 1.42 × 10^−6^; siNup153 WT versus siNup153 (GS)_4_-WT, *P* = 0.59. **l**, Directionality calculated as the ratio between the average import rate (top) and accumulation (bottom) for translocating R16-Ig27_Y9P_ versus Ig27_Y9P_-R16 constructs, measured when silencing individual FG-Nups. Error bars indicate s.e.m. **m**, Schematic of the proposed role of Nup153 in identifying locally unstructured protein regions. Significance levels for two-tailed *t*-test (**d**), two-tailed paired *t*-test (**e**), Mann–Whitney non-parametric test (**h**,**k**): NS, *P* > 0.05; *P < 0.05; **P < 0.01; ***P < 0.001; ****P < 0.0001. All points and bar plots indicate mean ± s.e.m.

To begin to investigate plausible molecular mechanisms underpinning the NPC sensitivity to the cargo mechano-directionality, we turned our attention to the FG-Nups (Fig. 5a), which represent about a third of nucleoporins^58–60^, fill the central channel of the NPC, and collectively create an effective and selective barrier for cargo translocation. We started by investigating Nup153, Nup98 and Nup214, which have been shown to play a particularly important role in nucleocytoplasmic transport^61–63^, despite being placed in different locations within the NPC, and have also been successfully used in biomimetic NPC systems^64^. It is particularly compelling that Nup153, while being in the nuclear basket, has been found to explore cytoplasmic regions of the NPC as well^65^, by potentially acting as a molecular spring that undergoes fast transitions between extended and collapsed conformations^66^. This rapid exchange between two separate locations within the NPC is suggestive of a Nup-mediated molecular motion mechanism able to facilitate transport across the pore^67^. Consequently, we conjectured that Nup153 might be involved in the mechanoselective transport of proteins across the NPC.

We first queried whether Nup153 specifically binds to protein cargos. We conducted a dot blot assay^68^, where Ig27_WT_ and (GS)_4_-Ig27_WT_ were immobilized on a nitrocellulose blotting membrane and then exposed to a bacterial cell lysate containing overexpressed StrepII-tagged FG-rich domains of Nup153, Nup214 and Nup98 (Fig. 5b). Our results demonstrate that Nup153 significantly binds Ig27_WT_, whereas Nup214 and Nup98 do not (Fig. 5c,d). It is noteworthy that Nup153 binds significantly (*P* = 0.04) better to the tagged (GS)_4_-Ig27_WT_ protein, suggesting a potential role of Nup153 in recognizing unstructured protein cargos (Fig. 5e).

**Extended Data Fig. 8:**
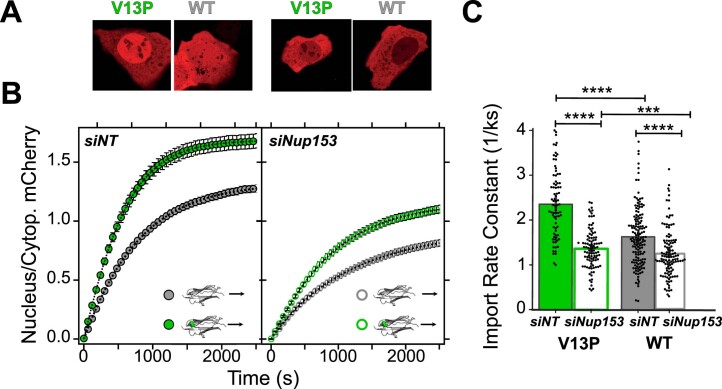
Knockdown of Nup153 decreases nuclear import kinetics but does not affect NPC mechanosensitivity. **(A)** Representative confocal images of U2OS cells 30 min in the recovery phase, scale bar 10 µm. **(B)** Average time courses of the relative nucleus-to-cytoplasm localization of V13P and WT under normal conditions (siNT) and Nup153 knockdown (siNup153). **(C)** Import rates calculated from fits to the recovery time courses. V13P (siNT) (*k*_I_ = 2.32 ± 0.09 ks^−1^*, n* = 82), V13P (siNup153) (*k*_I_ = 1.35 ± 0.04 ks^−1^*, n* = 100); WT (siNT) (*k*_I_ = 1.61 ± 0.05 ks^−1^*, n* = 147), WT (siNup153) (*k*_I_ = 1.22 ± 0.05 ks^−1^*, n* = 118). Significance levels for two-tailed Mann-Whitney non-parametric test NS > 0.05, *** P ≤ 0.001, **** *P* ≤ 0.0001. siNT V13P vs. siNup153 V13P, *P* = 3.21 × 10^−29^; siNT WT vs. siNup153 WT, *P* = 9.80 × 10^−10^; siNT V13P vs. siNT WT, *P* = 3.14 × 10^−11^. siNup153 V13P vs. siNup153 WT, *P* = 6.78 × 10^−5^. All points and bar plots indicate mean ± SEM.

We then tested whether these biochemical evidences would provide a molecular basis for the cellular translocation experiments by directly probing the effect of transiently silencing Nup153 with small interfering RNA (siNup153) on the kinetics of nuclear accumulation of the Ig27_WT_ and Ig27_V13P_ mutant (Extended Data Fig. 8). In both cases, we observed a marked decrease in nuclear entry upon Nup153 depletion, suggesting a clear role of Nup153 in regulating transport. However, the relative decrease in the rate of nuclear import with siNup153 was maintained for both proteins of different mechanical stabilities, suggesting that Nup153 does not dictate the mechanoselectivity of the NPC. To explore whether it affected the NPC sensitivity to the cargo mechano-directionality instead, we tested the effect of Nup153 depletion on the R16-Ig27_WT_/Ig27_WT_-R16 and R16-Ig27_Y9P_/Ig27_Y9P_-R16 construct pairs (Fig. 5f–h). Surprisingly, we observed that, in both cases, the mechano-directionality effect disappeared, entailing that, in the absence of Nup153, the rate of nuclear entry for each pair of constructs was independent of the mechanical stability of the leading protein. The same effect was observed when comparing the Ig27_WT_/(GS)_4_-Ig27_WT_ protein pair after Nup153 silencing (Fig. 5i–k), resulting in the abrogation of the GS-tag acceleration effect. Noteworthy, this effect was maintained upon exposing cells to amphiphilic agents such as *trans*-1,2-cyclohexanediol (CHD)^60^ and Pitstop-2^69^, which are known to disrupt FG-Nup interactions (Supplementary Figs. 8 and 9). Moreover, silencing Nup153 did not abrogate the NPC sensitivity to the cargo (R16-Ig27_Y9P_/Ig27_Y9P_-R16) mechano-directionality during export (Supplementary Fig. 10), hence restricting this particular mechanosensitive function of Nup153 to protein import.

To investigate whether other FG-Nups exhibit a similar mechanosensitive function, we repeated the optogenetic import experiments using the R16-Ig27_Y9P_/Ig27_Y9P_-R16 protein pair upon systematically silencing the majority of the individual FG-Nups essential for maintaining the permeability barrier, namely Nup98^62,64,70^, Nup214^71,72^, Nup54, Nup62, Nup358, Nup50, Nup58 and Tpr^58,59^. Remarkably, our measurements did not show any noticeable effect on the NPC’s sensitivity to the mechano-directionality of the shuttling cargos induced by any of the other FG-Nups (Fig. 5l and Supplementary Figs. 11–19). These results suggest that Nup153 plays a fundamentally specific and unique role in determining the NPC’s ability to sense the mechano-directionality of translocating proteins, probably by recognizing unstructured, mechanically labile, disordered protein regions as a first step during protein translocation across the NPC (Fig. 5m).

**Fig. 6:**
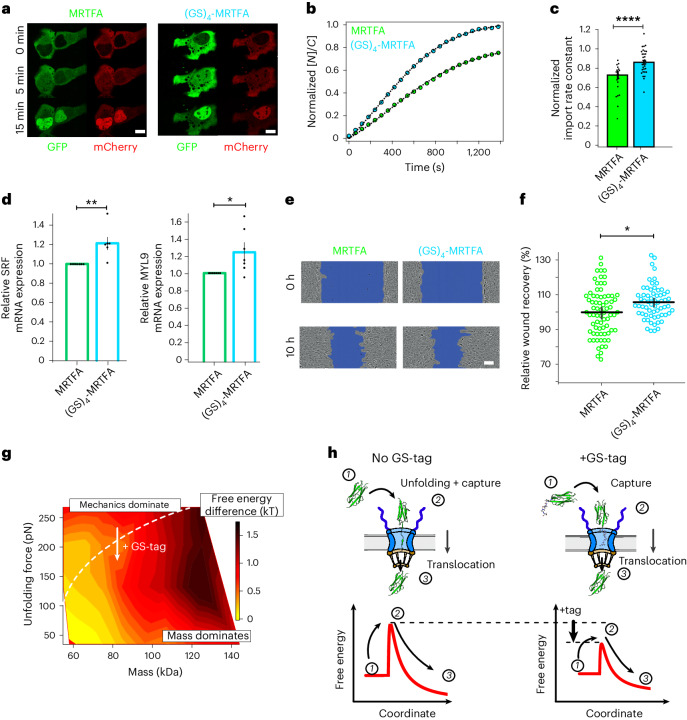
Mechanically labile peptide tags accelerate the nuclear import of the mechanosensitive myocardin-related transcription factor (MRTFA), resulting in increased gene expression and motility of U2OS cells. **a**, Representative confocal microscopy image gallery of U2OS doubly transfected with MRTFA-GFP–MRTFA-mCherry (left) and (GS)_4_-MRTFA-GFP–MRTFA-mCherry (right) at different time points after serum stimulation. Scale bars, 10 µm. **b**, Normalized average time courses of the relative nucleus-to-cytoplasm localization of MRTFA and (GS)_4_-MRTFA. The average time course is normalized by the average nucleus-to-cytoplasm accumulation (*K*_e_) of the co-translocating MRTFA-mCherry construct. **c**, Associated normalized nuclear import rates: MRTFA (${\widetilde{k}}_{\rm{I}}$ = 0.68 ± 0.03, *n* = 21); (GS)_4_-MRTFA (${\widetilde{k}}_{\rm{I}}$ = 0.87 ± 0.03, *n* = 34), where ${\widetilde{k}}_{\rm{I}}$ is the normalized import rate calculated as *k*_I_(GFP)/*k*_I_(mCherry). Significance levels for the two-tailed Mann–Whitney non-parametric test: ****P < 0.0001; MRTFA versus (GS)_4_-MRTFA, *P* = 3.33 × 10^−5^. **d**, RT–qPCR in U2OS cells stably expressing the MRTFA-GFP and (GS)_4_-MRTFA-GFP, 4 h after serum stimulation (*N* = 7 independent experiments). Bars show mean ± s.d. Significance levels for the two-tailed paired *t*-test after log transformation: *P < 0.01; **P < 0.001; SRF, *P* = 7.16 × 10^−3^, MYL9, *P* = 0.04. **e**,**f**, U2OS cells stably expressing GS-tagged MRTFA exhibit increased cell motility: representative wound-healing assays on U2OS stable cell lines expressing MRTFA or (GS)_4_-MRTFA (scale bar, 100 µm) (**e**); relative wound-healing recovery calculated from the velocity of the cell front during the first 10 h for MRTFA (*n* = 74) and (GS)_4_-MRTFA (*n* = 62) (**f**). Significance levels for the two-tailed *t*-test: *P < 0.05 (*P* = 0.03). **g**, Free-energy surface for active nucleoplasmic transport projected against the molecular weight and the unfolding force (characterized by AFM) of the translocating protein. The free-energy difference was calculated as ${\Delta G(M,\,{F}_{\rm{U}})}={-{kT}\,\mathrm{ln}\frac{{k}_{\rm{I}}(M,\,{F}_{\rm{U}})}{{k}_{\rm{I}}^{\rm{R}16}}}$, where *k*_I_ is the import rate for a protein of mass *M* and an unfolding force of *F*_U_, and where the import rate of the R16 monomer *k*_I_^R16^ has been used as reference. The dotted white line separates the regimes where mechanics or mass dominate the kinetics of nuclear import. **h**, Cartoon representation describing the passage of protein cargos across the NPC, in the absence (left) and presence (right) of a mechanically labile peptide tag. This results in the lowering of the free energy barrier and therefore in faster nuclear import kinetics. All points and bar plots indicate mean ± s.e.m.

**Extended Data Fig. 9:**
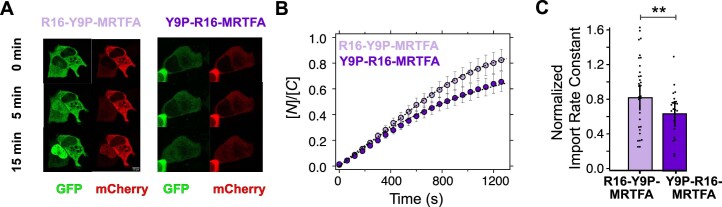
Mechano-directionality in the nuclear import of mechanosensitive myocardin-related transcription factor (MRTFA). **(A)** Representative confocal microscopy image gallery of U2OS doubly transfected with R16-Y9P-MRTFA-GFP – MRTFA-mCherry (left) and Y9P-R16-MRTFA-GFP – MRTFA-mCherry (right) at different time points after serum stimulation, scale bar 10 µm. The mCherry-tagged MRTFA is used for normalizing the nuclear import kinetics in each individual cell. **(B)** Normalized average time courses of the relative nucleus-to-cytoplasm localization of R16-Y9P-MRTFA and Y9P-R16-MRTFA. The average time course is normalized by the average nucleus-to-cytoplasm accumulation (*K*_*e*_) of the co-translocating MRTFA-mCherry construct. **(C)** Associated normalized nuclear import rates, R16-Y9P-MRTFA (${\widetilde{k}}_{I}$ = 0.90 ± 0.08*, n* = 35); Y9P-R16-MRTFA (${\widetilde{k}}_{I}$ = 0.70 ± 0.06*, n* = 25), where ${\widetilde{k}}_{I}$ is the normalized import rate calculated as *k*_*I*_(GFP)/*k*_*I*_(mCherry). Significance levels for two-tailed Mann-Whitney non-parametric test **P ≤ 0.01, *P* = 4.45 × 10^−3^. All points and bar plots indicate mean ± SEM.

Given that our findings focused on artificially devised cargo systems, we wondered whether the newly uncovered fundamental rules could serve as lessons to inspire the molecular engineering of naturally occurring proteins so as to rationally modify their dynamics of nuclear shuttling. We hence introduced the (GS)_4_ tag into the N-terminus of a green fluorescent protein (GFP)-tagged MRTFA^73^, close to its bipartite NLS sequence^74^. In U2OS doubly transfected with (GS)_4_-MRTFA-GFP and MRTFA-mCherry constructs (to provide an internal normalization in each cell), we found that the (GS)_4_-tagged MRTFA variant exhibited a significantly (*P* = 5 × 10^−5^) higher import rate and nuclear accumulation than the unmodified MRTFA (Fig. 6a–c). We also confirmed that (R16-Ig27_Y9P_)-MRTFA exhibits a significantly higher import rate than (Ig27_Y9P_-R16)-MRTFA, supporting our findings on the mechano-directionality of the translocating cargo (Extended Data Fig. 9). We then tested whether the enhanced nuclear accumulation of (GS)_4_-MRTFA with respect to the bare MRTFA could trigger genetic and functional knock-on effects. Because MRTFA is involved in the expression of genes related to adhesion and motility^75,76^, we conducted real-time quantitative polymerase chain reaction (RT–qPCR) experiments on stable U2OS cell lines expressing MRTFA-GFP and (GS)_4_-MRTFA-GFP and measured in each case, 4 h after serum stimulation, the levels of MYL9 and SRF messenger RNA (Fig. 6d). We found that cells stably expressing (GS)_4_-MRTFA-GFP exhibited significantly higher levels of *MYL9* (25% increase, *P* = 0.044) and *SRF* (23% increase, *P* = 0.007) mRNA expression compared with a MRTFA-GFP stable cell line. Finally, we performed a classical cell motility wound-healing assay that showed that U2OS cells stably expressing (GS)_4_-MRTFA closed a wound significantly faster than their MRTFA counterparts (Fig. 6e,f).

Although the biochemical mechanisms at play during nucleocytoplasmic transport have been investigated in depth, the role that the translocating cargo plays in determining its shuttling kinetics has attracted comparatively less attention. We previously reported that, in addition to molecular weight and exposed sequence, the mechanical stability of proteins emerges as an additional property to regulate nuclear trafficking dynamics^39^. Here we have extended these initial proof-of-principle studies by analysing the interplay between mass and mechanics. By systematically increasing the mass of the protein, we have found that proteins follow a ‘sequential’ scaling or ‘mass’ law when entering the NPC; however, strong deviations emerged at a certain threshold mass value, below which the mechanical stability—and not its mass—dominates nuclear import kinetics (Fig. 6g). That naturally led us to conjecture a direct relationship between the localized mechanical stability of a polyprotein (ultimately resulting from its local structure) and its nuclear transport dynamics. Specifically, we have demonstrated that mechanically labile, locally unstructured protein regions in the vicinity of the NLS increase the protein’s nuclear import rate across the NPC. These findings enable us to add the role of the protein cargo—based on the general physical principles regulating the translocation of polymers through a pore^77^—into the current description of the energy landscape governing protein traffic across the NPC. This view is based on the thermodynamic description of the ‘entropic exclusion’ model^78^—mostly centred on the interplay between reducing the conformational entropy of the FG-Nnups as cargos enter the pore (which increases with cargo size) and the attractive term stemming from the hydrophobic interactions between the FG-Nups and the translocating cargo, which counteracts the entropic penalty, reducing the total free energy and making cargo passage favourable. (Fig. 6h). The experiments presented here suggest that the physicochemical properties of the translocating protein cargo (namely its mass and its local mechanical stability) should be considered in this energetic treatment. From the cargo perspective, we propose that the first step in NPC translocation involves the capture of the protein by the NPC mouth. Such an initial capture process has an associated free-energy barrier that results from the entropic contribution of narrowing the conformational space and the energy penalty related to the creation of a mechanically unfolded protein segment that helps position the protein cargo for translocation. Crucially, the presence of either an engineered soft protein such as R16 (Fig. 3) or a mechanically labile tag such as a GS polypeptide (Fig. 4) bypasses the requirement of (partial) mechanical unfolding, and acts as a molecular handle to translocate into the nucleus, hence substantially lowering the underpinning energy barrier. Regardless of the molecular origin of the locally loose protein structure, Nup153 specifically binds to these disordered regions, reflective of an underpinning attractive interaction potential that lowers the energy barrier to translocation. This is consistent, at least in part, with the directional motion reported for Nup153, involving successive cycles of collapse and release^67^, that might be able to overcome the presence of the other barrier of Nups forming the entropic brush. Other translocation scenarios are, of course, also possible. For example, it has also been reported that Nup153 creates a dense meshwork or ‘hydrogel’, compatible with a selective phase mechanism where Nups physically interact with each other^79^. The resulting protein meshwork is predicted to be ‘dissolved’^80^ by cargo complexes. We conjecture that the enhanced ability of Nup153 to specifically bind unstructured cargos will facilitate their penetration through the Nup meshwork, because, at least from a steric perspective, partially unfolded protein conformations are likely to exhibit enhanced capabilities to navigate through small pore constrictions.

Fundamentally, our results suggest that asymmetric transport seems to be a direct consequence of the mechanical asymmetry of the translocating polyproteins. It remains to be addressed whether local structural anisotropy occurring within an even smaller, single protein domain might also influence the protein’s (mechano)-directional transport.

In a broader context, the results presented here hold large similarities with the mechanisms of protein translocation through other—arguably less complex and structurally narrower—biological pores. For example, in the bacterial ClpXP and ClpAP proteases, an unstructured region of the protein substrate (a degradation tag, or ‘degron’, residing at the C-terminus of a natively folded protein) is required to initially engage the protein substrate in the pore mouth, before the ATPase ring begins to pull and mechanically unfold the protein to enable its translocation across the pore for degradation^44^. Notably, the length and composition of the degron tag precisely define the specificity and degradation kinetics^81^. Similarly, during mitochondrial import, proteins are usually preceded by targeting precursor sequences^82^. The efficiency of protein import depends on the targeting sequence as well as on the local mechanical resistance of the protein structures adjacent to the targeting tags^11,83^. Consequently, our results demonstrate that, similar to mitochondria or the bacterial proteolytic machinery, the admittedly much more complex NPC is also able to sense intrinsically disordered protein tags as well as the local mechanical stability of the protein adjacent to the tag (leading protein). However, in contrast to these examples where proteins are mechanically unfolded by specific ATP-dependent unfoldases, we still do not know the precise molecular origin of the force required to release the unstructured protein segments before translocation, where specific molecular machines have not been explicitly identified. We conjecture that the directional, spring-like^67^ motion of specific Nups (such as Nup153) might help capture unstructured and mechanically weak protein regions as they enter the pore. It is possible that, similar to the role played by surface-exposed hydrophobic residues in artificial cargos^35^, the newly unfolded protein region of the engineered mechanical tag might be able to favourably interact with the different Nups encountered while being threaded through the pore, thereby not only helping in the initial capture step, but also the subsequent translocation stage. Future systematic sequence optimization of the polypeptide tag might result in further enhancement of the cargo’s nuclear import rate. It is also plausible that the diminished steric hindrance when locally unfolded protein regions (or unstructured tags) adjacent to the NLS are present increase the binding affinity for Kapα:Kapβ, thus favouring the binding of Kapβ with the FG-repeats, ultimately resulting in enhanced nuclear import.

It is thus enticing to speculate that transcription factors that need to constantly cross the NPC have evolved to exhibit intrinsically disordered regions. In fact, it is intriguing that transcription factors (such as MRTFA, for which the full X-ray crystal structure is lacking) are statistically enriched^84^ with intrinsically disordered and flexible regions. Consequently, one might expect that, in general, transcription factors will exhibit low mechanical resistance. For example, YAP is mechanically weak, as revealed by single-molecule AFM experiments^38^. From the biological viewpoint, structurally disordered regions in transcription factors are emerging as key determinants to guide protein–protein interactions in transcriptional condensates, actively contributing to DNA-binding specificity^85^. It is appealing to conjecture that these protein regions might include, or be close to, the NLS sequence, underscoring yet another layer of nuclear transport-based functionality.

In short, our work resonates with the rapidly emerging data unravelling the structural and functional complexity of the NPC^16–18,29,45^ and provides a cargo perspective, complementing pore-centric studies^66,86,87^, on the regulation of nucleocytoplasmic transport at the nanoscale. More generally, our approach might be useful as a biotechnological tool to deliver a wide range of cargos to the cell nucleus.

## Methods

### Plasmid construct and polyprotein engineering

For the optogenetic experiments using the NLS-mCherry-LEXY, the original pDN122 plasmid (Addgene 72655 ref. ^42^) was modified to remove the BglII site and the multicloning site. A cassette containing an NheI-NLS-BamHI-Ig27-KpnI-EheI sequence was inserted into the modified pDN122 between the NheI and AjiI sites before mCherry to create NLS-Ig27-mCherry-LEXY, allowing any DNA sequence to be swapped into the vector using BamHI-KpnI sites. Cloning was achieved using FastDigest Enzymes (Thermo Fisher Scientific) and T4 DNA ligase (New England Biolabs) unless otherwise stated.

Polymers were engineered by restriction digest using compatible cohesive ends restriction enzymes BamHI and BglII, with KpnI. For the final constructs, the terminal domain BamHI-X-KpnI was inserted between BglII and KpnI in the cleaved plasmid. To create GS-tagged proteins, a cassette of varying lengths of GS peptides, including an N-terminal BamHI site and C-terminal BglII and KpnI sites, was inserted into the modified pDN122 vector. Subsequent polymers were engineered as previously described. Cloning was achieved using FastDigest Enzymes (Thermo Fisher Scientific) and T4 DNA ligase (New England Biolabs). For MRTFA translocation experiments, (GS)_4_ was inserted in front of the MRTFA sequence (MRTFA-GFP vector kindly provided by M. Vartiainen^88^), by Gibson cloning after an L92M modification on the original plasmid to produce (GS)_4_-MRTFA-GFP (Gibson Assembly Master Mix, New England BioLabs). Recombinant plasmids were transformed into TOP10 competent cells (Thermo Fisher Scientific). Selected colonies were grown in Luria broth (LB) supplemented with 100 μg ml^−1^ ampicillin (or kanamycin 50 μg ml^−1^ for MRTFA constructs) at 37 °C, and the plasmid DNA was purified on GeneJet purification columns (Thermo Fisher Scientific), according to the manufacturer’s instructions.

For single-molecule experiments, polyproteins were engineered as previously described into pQE80L (Qiagen) between the BamHI and KpnI restriction sites. For AFM, polymers were designed with two additional cysteine residues at the C-terminus. For magnetic tweezers (MT) experiments, constructs were subcloned into a modified pFN18a vector (Promega) engineered with the AviTag (Avidity) (sequence GLNDIFEAQKIEWHE). Polymers were inserted between the HaloTag at the N-terminus and the histidine tag adjacent to the AviTag at the C-terminus using BamHI and KpnI restriction sites. Recombinant plasmids were transformed into TOP10 competent cells (Thermo Fisher Scientific). Selected colonies were grown in LB supplemented with 100 μg ml^−1^ carbenicillin at 37 °C, and the plasmid DNA was purified on GeneJet purification columns (Thermo Fisher Scientific), according to the manufacturer’s instructions. DNA sequences were verified by GENEWIZ (Azenta Life Sciences) or Source BioScience.

For the dot blot assays, DNA sequences encoding the FG-rich domains of Nup153 (residues 901–1475), Nup214 (residues 1400–2090) and Nup98 (residues 1–480) with a StrepII tag (located at the N terminus for Nup153 and Nup214 and at the C-terminus for Nup98) were synthesized by GeneArt (Thermo Fisher Scientific). These sequences were designed to incorporate *NdeI* and *XhoI* restriction sites for subsequent insertion into the pET50b+ plasmid. The coding sequences for I27_WT_ and (GS)_4_-I27_WT_, all tagged with 6x His at the C-terminus, were also cloned into the pET50b+ vector using *NdeI* and *XhoI* as restriction sites.

### Polyprotein expression and purification

Constructs encoding for the polyproteins of interest were expressed in *Escherichia coli* T7 Express cells (New England Biolabs) and grown in LB supplemented with 100 μg ml^−1^ carbenicillin at 37 °C. For the polyproteins used in MT experiments, biotinylation of the avidin tag was performed either in vivo (by co-expression of pBirAcm in medium supplemented with 34 mg ml^−1^ chloramphenicol) or post-elution through a purified BirA ligase (both Avidity). After reaching an optical density at 600 nm (OD_600_) of 0.6, cultures were induced with 1 mM isopropyl-β-d-thiogalactopyranoside (IPTG) and, for in vivo biotinylation, 50 μM d-biotin was also added. Cultures expressing polyproteins were incubated in a shaker incubator for 16 h at 20 °C. Cells were collected by centrifugation at 3,000*g* for 20 min and bacterial pellets of 500 ml of culture were resuspended in 25 ml of lysis buffer containing 20 mM HEPES pH 7.5, 300 mM NaCl, in the presence of protease inhibitor cocktail tablets (Roche), supplemented with 0.8 mg ml^−1^ lysozyme, 8 µg ml^−1^ DNase, 8 µg ml^−1^ RNase, 4 mM phenylmethylsulfonyl fluoride and 10 mM MgCl_2_. After incubation on ice for 30 min, the cells were disrupted by a French press (G. Heinemann) and the lysate was cleared by centrifugation at 39,000*g* for 45 min. The supernatant containing the soluble His-tagged protein was filtered with a cell strainer (40-µm filter) and then mixed with a HisPur Cobalt resin (Thermo Fisher Scientific) previously equilibrated in a buffer containing 20 mM HEPES pH 7.5 and 300 mM NaCl (buffer A) for 1 h at 4 °C under rotation.

The lysate/cobalt resin mixture was washed with 100 column volumes with buffer A containing 10 mM imidazole. Protein elution was carried out with buffer A containing 500 mM imidazole. The eluted fractions were collected and analysed by SDS–PAGE.

Fractions containing the protein were then collected and dialysed overnight against a buffer containing 20 mM HEPES pH 7.5 and 150 mM NaCl. The protein sample was concentrated using a Vivaspin centrifugal device (Sartorious) and further purified on a Superdex 200 Increase 10/300 GL column (Cytiva) using buffer A supplemented with 10% glycerol as running buffer. Elutions were analysed by SDS–PAGE, and pure protein samples were flash-frozen and stored at −80 °C.

For the dot blot assay, plasmids encoding for I27_WT_-6His and (GS)_4_-I27_WT_ -6His as well as the StrepII-tagged FG-rich domains of Nups 153, 214 and 98 were transformed into *E. coli* T7 Express cells (New England Biolabs) and cultured in LB medium supplemented with 50 µg ml^−1^ kanamycin at 37 °C. When the cultures reached an OD_600_ of ∼0.6, they were induced with 1 mM IPTG and allowed to grow at 20 °C for 16 h.

The optical densities (ODs) of cell cultures expressing the StrepII-tagged FG-rich domains of Nups 153, 214 and 98 were measured after induction with IPTG, and an adjusted volume containing precisely 750 Optical Density Units for each culture was pelleted. Bacterial cultures were subsequently subjected to centrifugation at 3,000*g* for 20 min at 4 °C, and the pellets were diluted in 25 ml of binding buffer (buffer A). Cells were disrupted using a French press. I27_WT_-6His and (GS)4-I27_WT_-6His protein purifications were conducted as described above.

### Cell culture, stable and transient transfection, and siRNA transfection and generation of stable cell lines

U2OS (American Type Culture Collection), NIH 3T3 (Francis Crick Institute) and HeLa (Francis Crick Institute) cells were grown in Dulbecco’s modified Eagle medium (DMEM), high glucose (Merck), supplemented with 10% fetal bovine serum (Merck) (for U2OS and HeLa cells) or 10% calf bovine serum (Merck) (for NIH 3T3 cells), 100 U ml^−1^ penicillin, 100 mg ml^−1^ streptomycin and 2 mM glutamine (Invitrogen). For transient protein expression, cells were transfected with 1 μg of constructs using HD FuGENE (Promega) according to the manufacturer’s protocol. To produce stable cell lines of MRTFA-GFP and (GS)_4_-MRTFA-GFP in U2OS, transiently transfected cells were selected with 500 μg ml^−1^ geneticin (Thermo Fisher Scientific), following fluorescence-activated cell sorting. Polyclonal cell lines were obtained and cultured in Complete medium supplemented with 500 μg ml^−1^ geneticin. For protein knockdown experiments, siRNA (non-targeting; D-001810-10-05, Nup153 (L-005283-00-0005), Nup98 (L-013078-00-0005), and Nup214 (L-011980-00-0005), Tpr (L-010548-00-0005), Nup62 (L-012468-00-0005), Nup50 (L-012369-01-0005), Nup58 (L-013864-01-0005), Nup358 (L-004746-00-0005), Nup54 (L-017570-01-0005); Horizon Discovery) was transfected into cells with HiPerFect (Qiagen) using the manufacturer’s protocol.

For MRTFA double transfection, cells were transfected with 1 μg of constructs (in total) using HD FuGENE (Promega) according to the manufacturer’s protocol. Cells were starved by withdrawing serum and incubating in a serum-starved medium containing 0.3% fetal bovine serum for 24 h. Starved cells were stimulated with 15% serum before imaging.

### Drug treatments

Pitstop-2 (abcam, ab120687) or dimethylsulfoxide (DMSO; control group) was added to the cells 30 min before image acquisition at a concentration of 30 µM, or, for DMSO, a concentration of 1:1,000. Samples were then imaged as described above. We added *trans*-1,2-cyclohexanediol (CHD; Merck, 141712) at a concentration of 20 mM 30 min before image acquisition. Saline buffer was added 30 min before image acquisition for the controls.

### Protein extraction and immunoblotting

Cells were lysed using radioimmunoprecipitation assay buffer with a protease inhibitor cocktail. The protein concentration of the lysates was determined with a Pierce BCA assay (Thermo Fisher Scientific), following separation by SDS–PAGE and transferal to a nitrocellulose membrane. For TPr and Nup358(RanBP2), NuPAGE 3–8% Tris-acetate gel (23060670, Thermo Fisher) was used, and Mini-PROTEAN TGX gel (4561096, Bio-Rad) was used for all other Nups. Membranes were incubated in blocking buffer (5% milk in TBS containing 0.2% Tween-20) for 1 h at room temperature and then incubated overnight with antibodies against Nup153 (A301-788A-T, Bethyl Laboratories), Nup98 (ab125980, Abcam), Nup214 (A300-717A-T, Bethyl Laboratories), Tpr (A300-828A, Bethyl Laboratories), RANBP2 (nup358) (16232-1-AP, Proteintech), Nup54 (27606-1-AP, Proteintech), Nup62 (13916-1-AP, Proteintech), NupL1 (Nup58) (19907-1-AP, Proteintech), Nup50 (20798-1-AP, Proteintech) and glyceraldehyde-3-phosphate dehydrogenase (GAPDH, Abcam). Antibodies were visualized using a chemiluminescence detection system (Bio-Rad). Densitometric analysis was performed using ImageJ.

### Wound-healing assay

U2OS cells stably expressing MRTFA-GFP and (GS)_4_-MRTFA-GFP were seeded onto ImageLock 96-well plates, which allow wound position tracking using Incucyte Zoom (Essen Bioscience). At 20 h before the beginning of the assay, the cells were serum-starved (0.3% serum). Scratch wounds were made using Woundmaker 96 (Essen Bioscience). The plate was washed once with phosphate-buffered saline (PBS), to remove any non-attached cells, then the cells were stimulated by the addition of cell medium containing 10% serum. Image acquisition and wound-length measurements from the acquired images were performed with Incucyte software. The closure of the wound was followed for 24 h (acquisition every 30 min). The wound-healing velocity was estimated from a linear fit to the first 10 h of the wound length versus time evolution. Final values were normalized with respect to MRTFA-GFP. Data were collected from six independent experiments.

### Live-cell image acquisition

Before image acquisition, HEPES pH 7 was added (to a final concentration of 50 mM) to the cells. Live-cell imaging was performed in an enclosed environment chamber (37 °C) with a confocal Nikon A1R inverted microscope with a ×60 NA 1.40 oil-immersion objective. Up to six sample positions were recorded for at least three independent replicates. The microscope was operated with the Nikon Perfect Focus System and controlled by NIS Elements software.

For optogenetic polyprotein experiments, image acquisition was performed throughout the activation and recovery phases. Activation of the NLS-X-mCherry-LEXY constructs was performed with a constant blue light (488 nm) illuminating the sample (10 min, a frame every min). The duration of the recovery period was increased with the number of protein domains in the construct, as higher molecular weights are associated with lower translocation rates and require longer observation times for accurate quantification (Supplementary Table 1).

For the MRTFA serum-stimulated experiments, the cells were incubated in serum-starved medium (0.3% serum) 16–20 h before the experiments. MRTFA translocation was triggered by the addition of 15% serum. Live-cell imaging was performed in an enclosed environment chamber (37 °C) with a confocal Nikon A1R inverted microscope with a ×60 NA 1.40 oil-immersion objective and laser and emission filter wavelengths of 488.2 nm and 540/30 nm for GFP and 561.9 nm and 595/50 nm, respectively for mCherry. Images were typically acquired every 60 s over 45 min.

To study protein export, we used a related optogenetic construct, NES-X-mCherry-AsLOV2-NLS, where the position of the NES and NLS tags has been swapped. In this construct, upon blue-light exposure, the protein cargo shuttles into the nucleus. Under dark conditions, the NES tag excludes the protein from the nucleus, hence increasing the cytoplasm/nucleus ratio, enabling direct quantification of the export rate. To activate protein export, cells were exposed to constant blue-light illumination (20 min, frame every min). The duration of the recovery period for the tested export constructs was 45 min, acquiring one image frame per minute.

### RNA extraction and qPCR

RNA was extracted from U2OS cells using an RNeasy Mini kit (Qiagen) according to the manufacturer’s instructions, and was then treated with DNase (Invitrogen). The RNA concentration was determined and the purity checked by measuring the ratio of absorbance at 260 and 280 nm. RNA was converted to complementary DNA (cDNA) using iScript reverse transcription supermix for RT–qPCR (Bio-Rad) according to the manufacturer’s instructions. The relative mRNA expression of genes was determined by RT–qPCR assay using SYBR-Green detection chemistry (Applied Biosystems) and a QuantStudio 3 RT–qPCR system (Thermo Fisher). The primers used are detailed in Supplementary Table 2. The relative abundance of template cDNA was calculated by the comparative CT (ΔΔCT) method. Each sample was normalized to the level of GAPDH.

### Dot blot assay

Four small nitrocellulose blotting membranes (Amersham Protran 0.2-µm NC) were cut into quadrangular shapes, each measuring ~5.5 × 5.5 cm. The purified proteins were diluted to immobilize an equivalent amount of I27_WT_-6His and (GS)_4_-I27_WT_-6His, respectively. Spots containing these purified proteins were then fixed in triplicate onto three respective rows for each membrane and dried at room temperature for ~20 min.

The control membrane was blocked in a Petri dish with 5% (wt/vol) dry skimmed milk in TBS solution with 0.1% Tween (TBS-T), and the other three were blocked with 3% bovine serum albumin (BSA) in PBS-T (0.5% Tween in PBS) for a duration of 16 h at 4 °C.

The blots were washed twice for 5 min in TBS-T and PBS-T, respectively. Subsequently, they were incubated overnight at 4 °C with 750 OD units of bacterial lysates from cultures containing the overexpressed StrepII-Nup153, StrepII-Nup214 and StrepII-Nup98, respectively. The control membrane was incubated with the binding buffer.

The membranes were then washed three times in TBS-T and PBS-T, respectively. Afterwards, they were incubated at room temperature for 1 h with the corresponding antibodies. On the control membrane, horseradish peroxidase (HRP)-conjugated anti-His-Tag antibody was used at a 1:5,000 dilution in TBS-T. For the other three membranes, the Strep•Tag II antibody HRP conjugate was used at a 1:4,000 dilution in PBS-T to detect the binding of Nup to immobilized proteins.

Six additional washes were completed before membrane development, which was performed using a SuperSignal West Pico chemiluminescent detection kit from Thermo Scientific, following the manufacturer’s recommendations.

To quantify the dot blot assay, dot blot intensity was calculated as the pixel intensity (in black) of the dot region after substracting its background. For each experiment (membrane), the dot blot intensity for a particular group (for example, Ig27_WT_- with Nup153) was calculated as the average intensity of the three dots normalized by the average intensity of the three dots in the corresponding control membrane (for example, Ig27_WT_-). A total of five of five dot blot experiments were conducted.

### Live-cell quantification

Image quantification was performed using a custom-made MATLAB script. The image collections for each recorded position were initially background-corrected, and then the total fluorescent intensities of the nucleus and cell were defined manually. For each frame, the nuclear or cytoplasmic signals were divided by the total cellular signal, and this quantity was used as a proxy for the nuclear or cytoplasmic protein concentrations, respectively. To quantify the nuclear import kinetics, we assumed a simple first-order kinetic process. For large LEXY constructs (>45 kDa), (1) passive transport is negligible and (2) there is no active export, because the NES sequence docked onto the LOV domain and is not accessible to the exportins^42^. Therefore, the recovery kinetics arise from the NLS-driven active import of the protein cargo into the nucleus. That way, the nuclear protein concentration [*N*](*t*) will increase in time as

$${[N\;](t)}={{[N\;]}_{\rm{e}}-({[N\;]}_{{\rm{e}}}-{[N\;]}_{0}){\rm{e}}^{-{\rm{k}}_{{\rm{I}}}{\rm{t}}}}$$

where [*N*]_0_ and [*N*]_e_ are the initial and steady-state nuclear concentrations, respectively, and *k*_I_ is the import rate constant. Similarly, the cytoplasmatic concentration will evolve as

$${[C](t)}={{[C\;]}_{{\rm{e}}}+({[C\;]}_{0}-{[C]}_{{\rm{e}}}){\rm{e}}^{-{\rm{k}}_{{\rm{I}}}{\rm{t}}}}$$

Because the nucleus and cytoplasm have different volumes (and therefore the same number of proteins in the nucleus or in the cytoplasm will correspond to a different concentration), we can define the nucleus-to-cytoplasmatic volume ratio as

$${v}={\frac{{V}_{{{\rm{N}}}}}{{V}_{{{\rm{C}}}}}=\frac{{[C]}_{0}-{[C]}_{{\rm{e}}}}{{[N]}_{{\rm{e}}}-{[N]}_{0}}}$$

because the total number of proteins is constant. Our initial conditions correspond to the end of the activation phase, during which blue light was applied for 10 min, releasing the docked NES sequence and triggering mobilization of the protein construct out of the nucleus. Due to the much higher strength of the NES sequence with respect to the NLS one, the activation phase is dominated by the export kinetics, with a negligible contribution of active import^42^. Therefore, we can assume [*N*]_0_ → 0; [*C*]_0_ → [*C*]_0_ + *v*[*N*]_0_, which allows us to write the time course of the nucleus-to-cytoplasm protein concentration as

1 $${\frac{\left[N\;\right]}{\left[C\right]}\left(\rm{t}\right)}={\frac{{K}_{\rm{e}}\left(1-{\rm{e}}^{-{\rm{k}}_{\rm{I}}\rm{t}}\right)}{\left(1+{v{K}_{{\rm{e}}}{\rm{e}}}^{-{\rm{k}}_{\rm{I}}\rm{t}}\right)}}$$

where *K*_e_ = [*N*]_e_/[*C*]_e_ is the relative nucleus-to-cytoplasm accumulation in the steady state. Importantly, *K*_e_ and *k*_I_ are two uncoupled (free) parameters because there is no active protein export that contributes to the relaxation to the steady state. Although in most of the measured protein constructs *K*_e_ and *k*_I_ appear correlated (a faster import rate is typically associated with a higher nuclear accumulation, although there are some notable exceptions), both parameters have a different physical meaning: *k*_I_ is the rate constant that defines the import kinetics into the nucleus, while *K*_e_ represents the fraction of mobile proteins, related to the efficiency of the transport process, and is also construct-dependent.

In the case of MRTFA, by contrast, active export is not negligible due to its NES sequence. Although typically the export rate is much lower than the import rate under serum stimulation (~10%)^39^, one should always explicitly account for the import and export rates in the kinetic model, which leads to the following expression for the relative nucleus-to-cytoplasm protein concentrations^39^:

2 $${\frac{\left[N\;\right]}{\left[C\;\right]}\left(t\right)}={\frac{{K}_{\rm{e}}\left(1-{\rm{e}}^{-{\rm{kt}}}\right)}{\left(1+{v{K}_{{\rm{e}}}{\rm{e}}}^{-{\rm{kt}}}\right)}}$$

Although, formally, both equations have the same shape, here *K*_e_ = *k*_I_/*k*_E_ and *k* = *k*_E_ + *vk*_I_.

We quantify the live-cell images by measuring the nuclear and cytoplasmic signals. By fitting the time course of [*N*]/[*C*] during the recovery phase to equation (1), we extract the relative nuclear accumulation *K*_e_ and import rate *k*_I_. At the start of the recovery phase, there is a cell-to-cell variation in the initial conditions, which we correct by fitting the raw nuclear signal to *n*_0_e^−kt^ + *n*_e_(1 *−* e^−kt^) and subtracting *n*_0_e^−kt^ from each data point (Supplementary Fig. 1a,b). We then fit the corrected [*N*]/[*C*](*t*) to equation (1) and extract *K*_e_ and *k*_I_, which characterize the import dynamics for that specific cell (Supplementary Fig. 1b). Spurious cell measurements are removed from the analysis by applying a semi-automatic three-step filtering protocol:

1. Unphysical fits and outliers are removed using an iterative algorithm (generalized extreme studentized deviate), with a tolerance of two standard deviations.
2. Poorly activated cells (not meeting the initial conditions requirement assumed in the model) are filtered by evaluating the export rate constant from a single exponential fit to the nucleus signal in the activation phase. If the obtained export rate constant is lower than 1.4 ks^−1^ (85% of the duration of the activation phase), the cell is assumed to be poorly activated and is discarded.
3. A human inspection of the cells that passed steps 1 and 2 is finally done to discard spurious data that escaped the automatic filtering steps.

For a given protein construct, we quantify its nuclear entry properties with the average time course of the nucleus-to-cytoplasm protein concentration (calculated as a point-by-point average of each single cell recovery curve; Supplementary Fig. 1c) and by the distributions of *K*_e_ and *k*_I_. Supplementary Fig. 1d shows the distributions of *K*_e_ (accumulation) and *k*_I_ (import rate constant) for the NLS-(Ig27_WT_)-mCherry-LEXY protein construct. To perform statistical comparisons between pairs of interest in LEXY (or MRTFA) experiments, we use a Mann–Whitney non-parametric test on the distribution of import rates *k*_I_, given their markedly non-Gaussian shape.

Analysis of nuclear export experiments was conducted in an analogous way, using in this case the cytoplasm-to-nucleus ratio ([*C*]/[*N*](*t*)) instead of the nucleus-to-cytoplasm ratio.

### ‘Stochastic’ versus sequential unfolding model

In a single-molecule force spectroscopy experiment, a polyprotein composed of *N* identical domains is stretched from the molecule termini, implying that all *N* domains are exposed to force. All domains will thus unfold stochastically following their unfolding rate *r*_U_, following first-order kinetics. According to this, the total rate to unfold the polyprotein *r*_*N*_ (this is the inverse of the mean-first-passage time to the last unfolding event) scales with the number of domains *N* as

$${r}_{\rm{N}}{(N\,)}={\frac{{r}_{\rm{U}}}{\mathop{\sum }\nolimits_{\rm{n=1}}^{\rm{N}}\frac{1}{n}}}$$

where this is a purely Markovian process.

By contrast, if a polyprotein unfolds sequentially, domain *i* can only unfold if domain *i* − 1 has previously unfolded, and the unfolding rate of the full polyprotein scales with the number of domains *N* as

$${r}_{N}{(N\;)}={\frac{{r}_{\rm{U}}}{N}}$$

### AFM force spectroscopy

Single-molecule AFM experiments were conducted using a Luigs and Neumann instrument operating at room temperature, as previously described^89^. During sample preparation, 0.5–2 μl of protein (1–5 mg ml^−1^ in PBS pH 7.3) was spread onto a gold-coated coverslip, previously plasma-cleaned for 10 min. Before each experiment, the cantilever (Si_3_N_4_ MLCT-C, Bruker) was calibrated using the equipartition theorem, obtaining a spring constant of ~12–20 pN nm^−1^.

In the force–extension mode, the experiment was initiated by first pressing the cantilever tip against the protein-coated surface with high force (∼1,000 pN) to achieve non-specific binding between a protein and the tip. Then, for both polyprotein constructs ((Ig27_WT_)_2_-(R16)_2_ and (R16)_2_-(Ig27_WT_)_2_), the piezo-mounted surface was retracted at a constant velocity of 400 nm s^−1^, resulting in application of an increasing force. Data were recorded and analysed using a custom-made software script in Igor Pro (WaveMetrics). During the recordings, only traces showing both Ig27_WT_ domains were selected for analysis. To obtain the increment in contour length, every protein unfolding event was fitted to the worm-like chain model of polymer elasticity. Unfolding forces were determined from the position of the peaks.

When studying the unfolding rate of (Ig27_WT_)_*N*_ at constant force (force-clamp mode), the experiment was begun by pressing the cantilever against the surface (∼500–2,000 pN for 1 s) to allow protein adhesion. The piezoelectric actuator was then retracted to achieve a constant force of 150 pN, maintained by an active feedback system that corrected the position of the piezo on a timescale of ∼1–5 ms to maintain a constant cantilever deflection of the cantilever (and hence a constant force). All force traces were filtered using a pole Bessel filter at 1 kHz. To analyse the force-clamp trajectories, only recordings showing ≥2 steps of 25 nm were selected to ensure a robust molecular fingerprint. Additionally, only recordings showing a detachment time three times longer than the Ig27 unfolding time were further analysed, to ensure that all Ig27 domains exposed to force had unfolded.

### MT force spectroscopy

Single-molecule MT experiments were conducted on a custom-made set-up, as described in ref. ^90^. Briefly, the set-up was built on an inverted microscope (Nikon) with the magnets (N52) mounted on a voice coil (Equipment Solutions) to control their vertical position, and placed on top of a ×100 oil-immersion objective (Nikon) mounted on a piezoelectric actuator (PI). Illumination was provided by a white light-emitting diode cold light source (Thorlabs), while image acquisition was achieved with a complementary metal–oxide–semiconductor camera (Ximea). Control of the magnet’s position and the piezo was achieved with a multifunction DAQ card (National Instruments), using custom-made data-acquisition software.

The molecule of interest (R16-Ig27_WT_ and Ig27_WT_-R16 in our case) was tethered to a superparamagnetic Dynabeads M-270 streptavidin-coated bead (Invitrogen), which typically resists forces up to 120 pN. The sample was prepared in custom-made fluid chambers consisting of two glass coverslips (Menzel–Glaser) separated by a laser-cut parafilm pattern. The fluid chamber was functionalized with HaloTag O4 ligand^90^ to achieve covalent and specific anchoring of the N-terminal HaloTag protein constructs, and amino-coated non-magnetic beads were used as reference beads. The chamber was passivated using Tris blocking buffer (20 mM Tris-HCl pH 7.4, 150 mM NaCl, 2 mM MgCl_2_ and 1% wt/vol sulfhydryl blocked BSA).

The protein was incubated in the fluid chamber for ∼30 min (1–5 nM) to ensure attachment, and the experiments were conducted in PBS containing 10 mM ascorbic acid pH 7.4 to minimize oxidative damage. M-270 beads (∼20 μl) were added in the chamber and incubated for 5 min before force application. To unfold the R16-Ig27_WT_ or Ig27_WT_-R16 constructs, a linear force ramp at a loading rate of 1 pN s^−1^ between 4 and 110 pN was applied. The observed step size for each event was converted to a contour length increment using the freely jointed chain model assuming a Kuhn length of 1.1 nm, so each event was characterized by its unfolding force and contour length increment. On every occasion, R16 unfolded first at a force of ∼25–30 pN (showing a step size of ∼21 nm that indicates a contour length increment of ∼35 nm) and Ig27_WT_ second at a force of ∼95–105 pN (showing a step size of ∼25 nm, indicating a contour length of ∼28 nm).

## Online content

Any methods, additional references, Nature Portfolio reporting summaries, source data, extended data, supplementary information, acknowledgements, peer review information; details of author contributions and competing interests; and statements of data and code availability are available at 10.1038/s41567-024-02438-8.

### Supplementary information

Supplementary Information Supplementary Figs. 1–19 and Tables.

## Data Availability

The data that support the plots within this paper and other findings of this study are available from the corresponding author upon reasonable request.

## Code availability

The code is available from the corresponding author upon reasonable request.
