# Supplementary material for: Structural anisotropy results in mechano-directional transport of proteins across nuclear pores
Source: Nat Phys. 2024 May 13;20(7):1180–93. doi: 10.1038/s41567-024-02438-8 (PMC11254768; doi:10.1038/s41567-024-02438-8)
Supplement: Supplementary file 1 — Supplementary Figs. 1–19 and Tables. [file 41567_2024_2438_MOESM1_ESM.pdf]

nature physics

Article

https://doi.org/10.1038/s41567-024-02438-8

Structural anisotropy results in mechano-directional transport of proteins across nuclear pores

In the format provided by the authors and unedited

Table of Contents

Supplementary Figures

Supplementary Tables

Supplementary Figures:

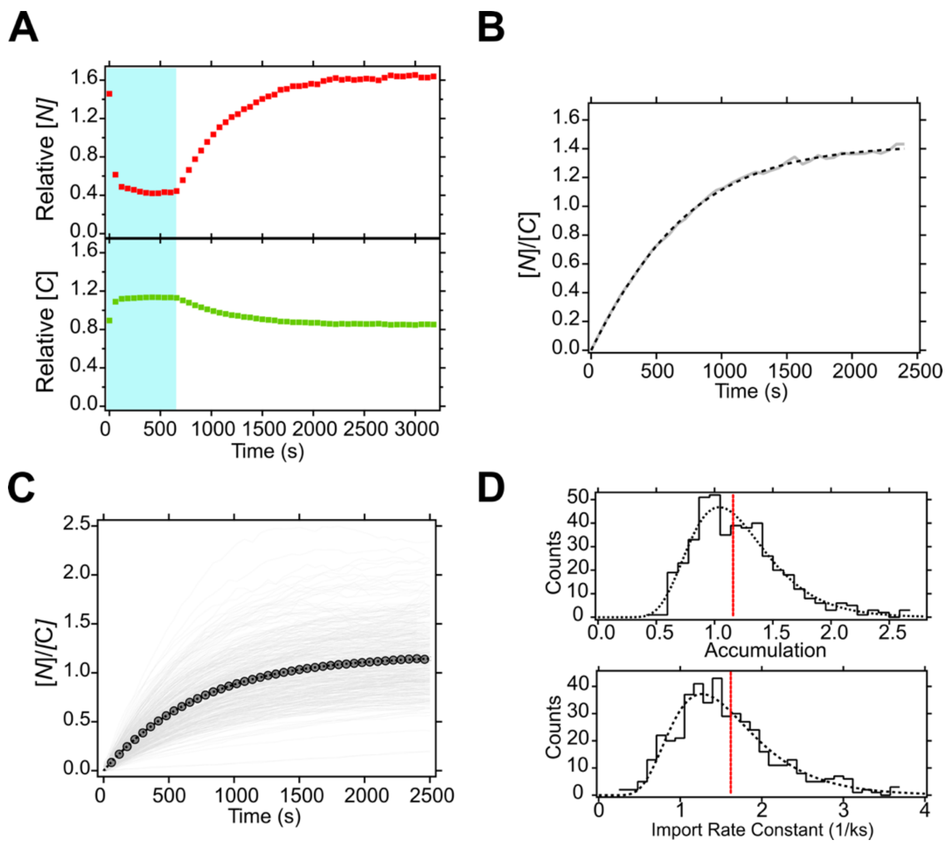

Supplementary figure 1. Data processing methodology. (A) Raw nucleus/cell (relative [*N*], upper, red) and cytoplasm/cell (relative [*C*], lower, green) time course localization of the NLS-Ig27$_{WT}$-mCherry-LEXY construct in a single cell experiment, used to exemplify our data processing methodology. During the 10 minutes of the activation phase (blue shaded region), the protein construct is rapidly mobilized out of the nucleus, which consequently increases its concentration in the cytoplasm. During the recovery phase, the NLS drives the import of the protein construct into the nucleus, concomitantly decreasing the relative cytoplasmic concentration. **(B)** Processed nucleus-to-cytoplasm mCherry signal of NLS-Ig27$_{WT}$-mCherry-LEXY for a single cell during the recovery phase ([*N*]/[*C*]). After the data-filtering protocol to discard spurious cells, we correct the cell-to-cell variation of the initial conditions by fitting the raw nucleus/cell signal to $n_0e^{-kt}+n_e(1-e^{-kt})$ and subtracting n_0e^{-kt} to each point. Fitting the corrected nucleus/cytoplasm data to ***Eq. 1***, allows us to extract the parameters characterizing the nuclear import kinetics (accumulation K_e, and import rate constant k_I) for that single cell. **(C)** Individual time courses of corrected nucleus/cytoplasm mCherry signals ([*N*]/[*C*]) for the NLS-Ig27$_{WT}$-mCherry-LEXY construct shown for *n*=435 cells. The average nucleus/cytoplasm time course is calculated as a point-by-point average of the single-cell time courses (gray circles), used as the representation of the average import kinetics for that protein construct. **(D)** Distribution of accumulation (K_e) and import rate constant (k_I) for NLS-Ig27$_{WT}$ -mCherry-LEXY as obtained from fits to *n*=435 cells. The parameters are not Gaussian-distributed (rather a log-normal distribution), which requires application of non-parametric statistical

tests to compare the kinetics of different protein constructs. The vertical red line shows the average value, which sits to the right of the peak due to the asymmetry of the distribution.

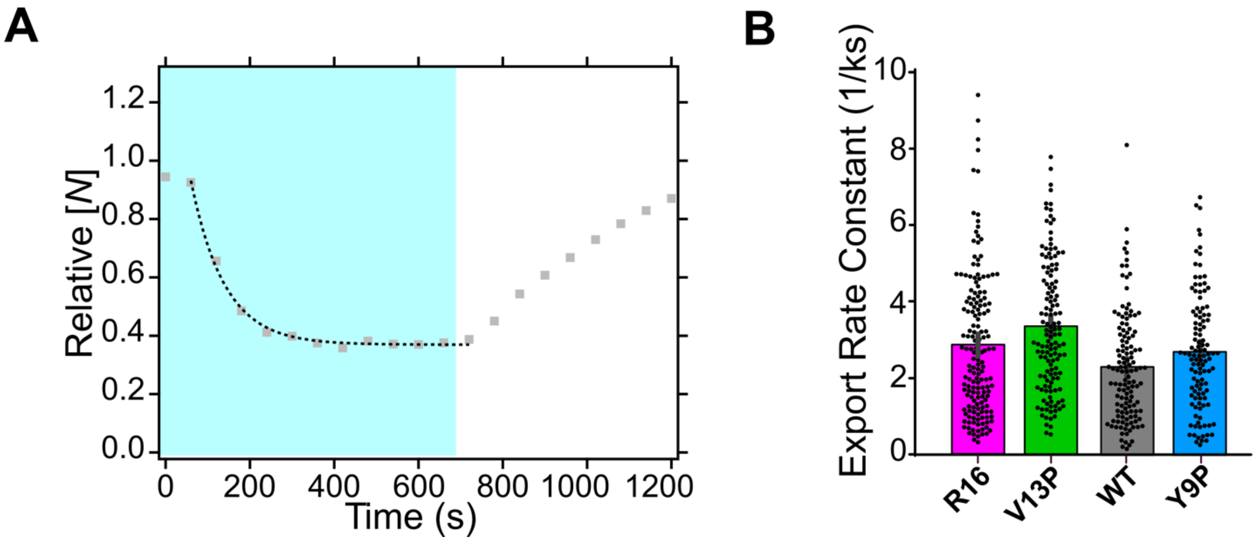

Supplementary figure 2. Quantification of the export kinetics from the activation phase: (A) Time course localization of a NLS-Ig27$_{WT}$-mCherry-LEXY construct within a single cell. The export kinetics during the activation phase is highlighted in blue. Exposure of the cell to blue light reveals the NES, rapidly mobilizing the protein cargo out of the nucleus. Fitting a single exponential allows extracting the effective export rate constant. **(B)** Export rate constant for the different monomers (R16, Ig27$_{V13P}$, Ig27$_{WT}$, Ig27$_{Y9P}$). In contrast to the import kinetics, there is no clear correlation between the export kinetics and the mechanical stability of the protein cargo.

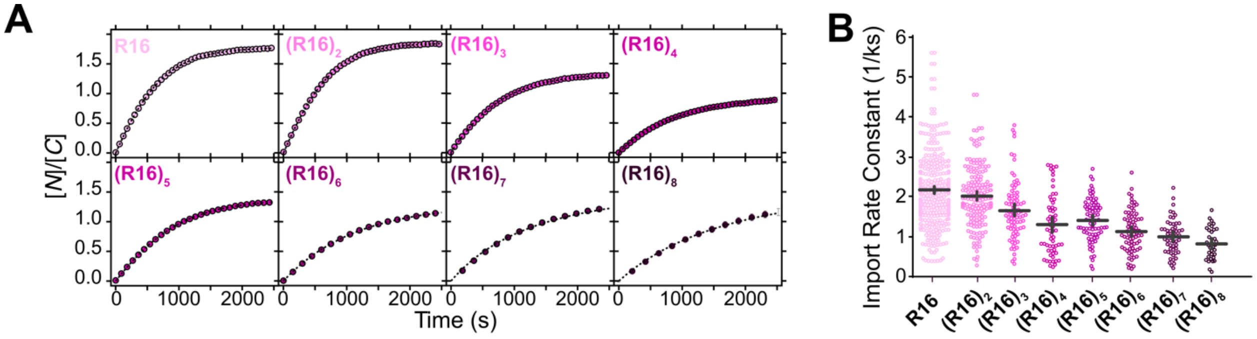

Supplementary figure 3. Translocation kinetics of the Spectrin R16 polyprotein. (A) Nucleus-to-cytoplasm time course localization of the NLS-$(R16)_X$-mCherry-LEXY construct, being X the number of R16 domains. **(B)** Import rate constants for the R16 polyproteins. Each construct was measured on three independent experiments, with *n*=213 (R16); *n*=184 $(R16)_2$; *n*=85 $(R16)_3$; *n*=62 $(R16)_4$; *n*=77 $(R16)_5$; *n*=77 $(R16)_6$; *n*=55 $(R16)_7$; *n*=35 $(R16)_8$. Horizontal bars indicate the average import rate constant, and vertical bars the SEM.

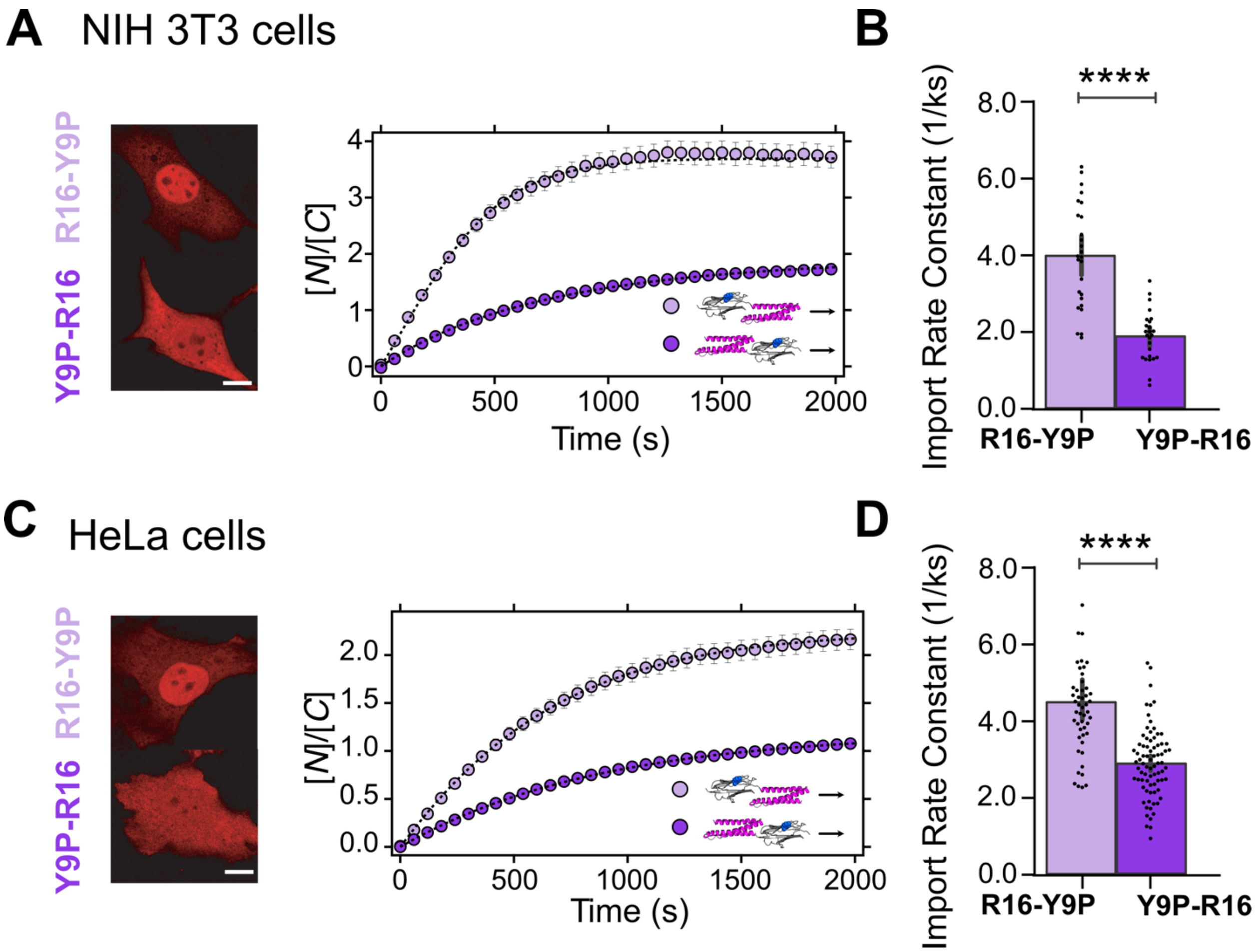

Supplementary figure 4. Effect of the mechanodirectionality of the protein cargo on its nuclear accumulation in NIH 3T3 and HeLa cells. (A) Left: Representative confocal images of NIH 3T3 cells 30 min in the recovery phase. Scale bar 10 µm. Right: Average time courses of the relative nucleus-to-cytoplasm localization of R16-Y9P and Y9P-R16 constructs. **(B)** Protein import rate constants (mean ± SEM) calculated from the accumulation curves. R16-Y9P (k_I=3.89±0.26 ks^{-1}, *n*=24), Y9P-R16 (k_I=1.85±0.13 ks^{-1}, *n*=25). Significance levels from two-tailed Mann-Whitney test. ****P<0.00001, P=3.34x10^{-7}. **(C)** Left: Representative confocal images of HeLa cells 30 min in the recovery phase. Scale bar 10 µm. Right: Average time courses of the relative nucleus-to-cytoplasm localization of R16-Y9P and Y9P-R16 **(D)** Protein import rate constants (mean ± SEM) calculated from the accumulation curves. R16-Y9P (k_I=2.22±0.13 ks^{-1}, *n*=49), Y9P-R16 (k_I=1.42±0.05 ks^{-1}, *n*=78. Significance levels from two-tailed Mann-Whitney test. ****P<0.00001, P=9.14x10^{-11}. All points and bar plots indicate mean±SEM.

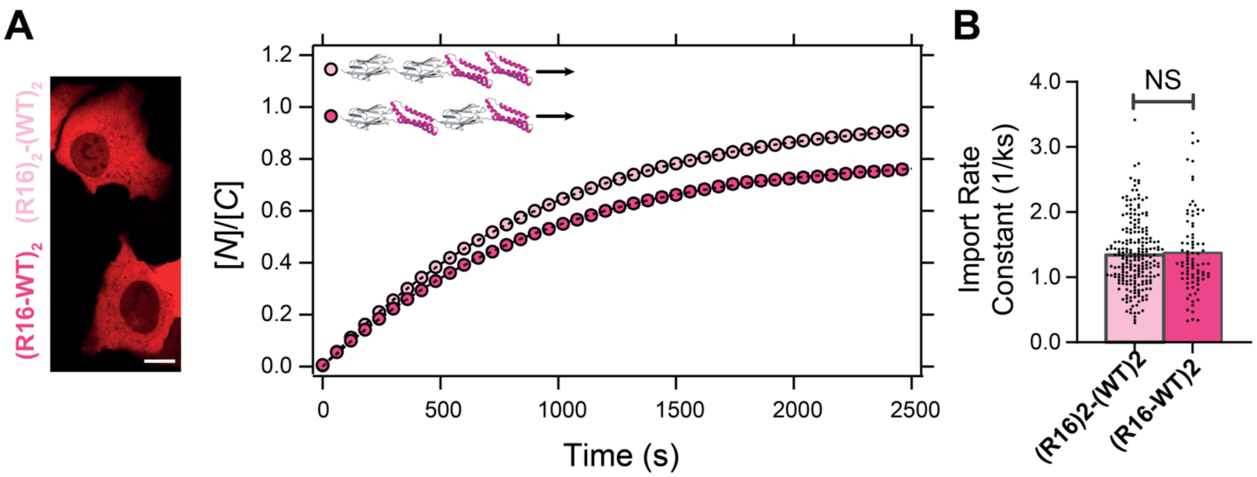

Supplementary figure 5. The nuclear import dynamics of a polyprotein are dictated by the mechanical stability of the N-terminal (leading) domain closer to the NLS sequence. (A) Left: Representative confocal images of U2OS cells 30 min in the recovery phase. Scale bar 10 µm. Right: Average time courses of the relative nucleus-to-cytoplasm localization of $(R16)_2$-$(WT)_2$ and $(R16\text{-}WT)_2$ protein constructs. **(B)** Protein import rate constants (mean ± SEM) calculated from the accumulation curves. $(R16)_2$-$(WT)_2$, (k_I=1.34±0.04 ks^{-1}, n=217); $(R16\text{-}WT)_2$, (k_I=1.38±0.07 ks^{-1}, n=83). Significance levels for Mann-Whitney non-parametric test NS $P > 0.05$. P=0.93. All points and bar plots indicate mean±SEM.

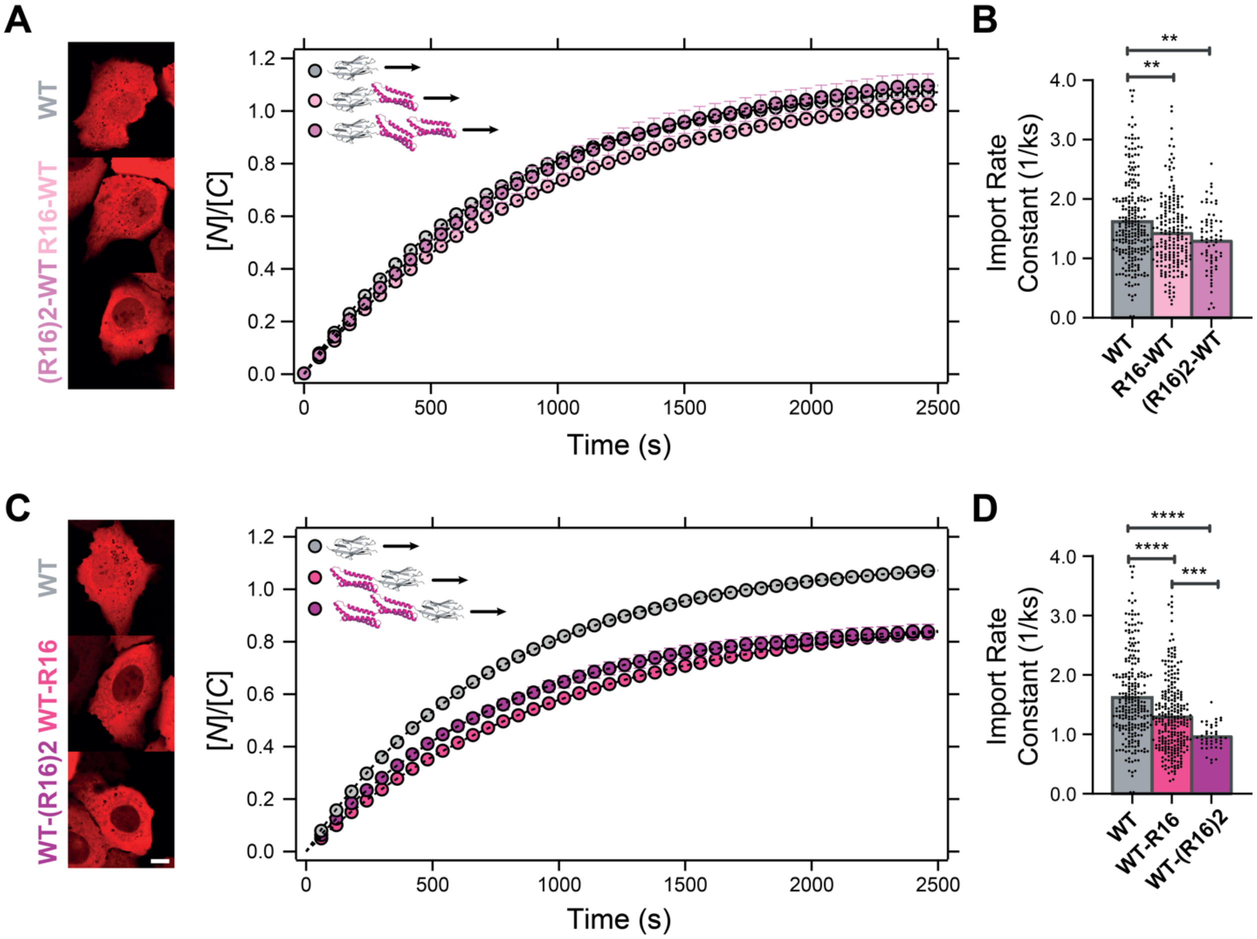

Supplementary figure 6. Addition of individual low mechanical stability protein domains close to the NLS of a stiffer protein cargo do not compromise its nuclear accumulation despite the mass increase. (A, C) Left: Representative confocal images of U2OS cells 30 min in the recovery phase. Scale bar 10 µm. Right: Average time courses of the relative nucleus-to-cytoplasm localization of (**A**) WT, R16-WT, $(R16)_2$-WT and (**C**) WT, WT-R16, WT-$(R16)_2$ protein constructs. **(B, D)** Protein import rates constants (mean ± SEM) calculated from the accumulation curves. WT (k_I=1.62±0.05 ks^{-1}, *n*=249); R16-WT (k_I=1.42±0.04 ks^{-1}, *n*=193); $(R16)_2$-WT (k_I=1.29±0.06 ks^{-1}, *n*=69); WT-R16 (k_I=1.29±0.04 ks^{-1}, *n*=259); WT-$(R16)_2$ (k_I=1.02±0.07 ks^{-1}, *n*=42). Significance levels for two-tailed Mann-Whitney non-parametric test ** $P \leq 0.01$, *** $P \leq 0.001$, **** $P \leq 0.0001$. WT vs. R16-WT, $P=3.84\times10^{-3}$, WT vs. $(R16)_2$-WT, $P=1.12\times10^{-3}$, WT vs. WT-R16, $P=6.99\times10^{-8}$, WT vs. WT-$(R16)_2$, $P=5.80\times10^{-10}$, WT-R16 vs. WT-$(R16)_2$, $P=1.89\times10^{-4}$. All points and bar plots indicate mean±SEM.

.

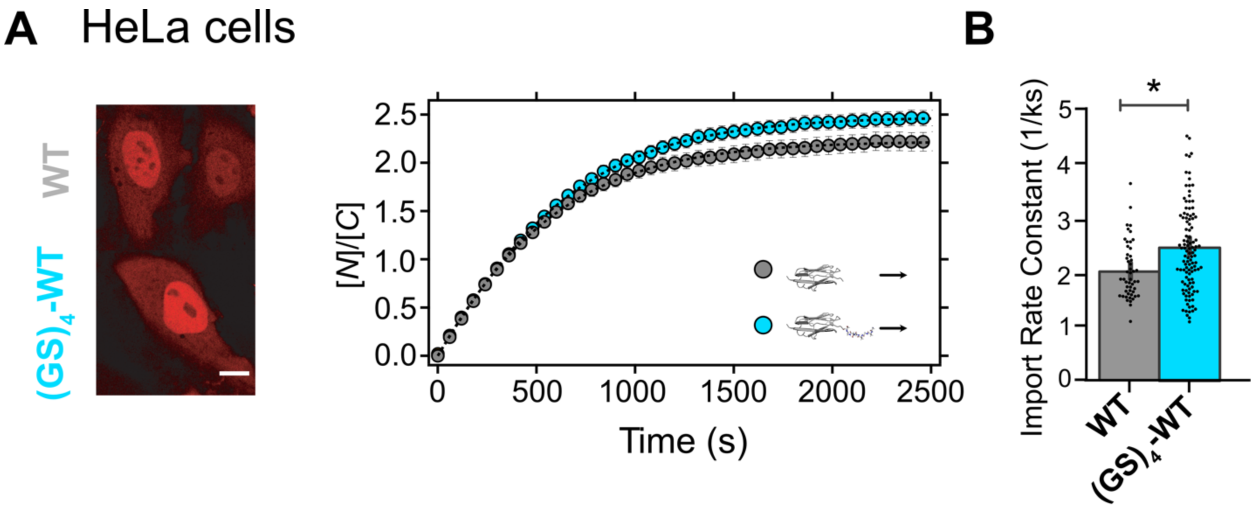

Supplementary figure 7. Addition of the unstructured (GS)$_4$ peptide in front of Ig27$_{WT}$ accelerated its nuclear import in HeLa cells. (A) Left: Representative confocal images of HeLa cells 30 min in the recovery phase. Scale bar 10 µm. Right: Average time courses of the relative nucleus-to-cytoplasm localization of WT and (GS)$_4$-WT, showing that the (GS)-tag also accelerates the nuclear import of protein cargos in HeLa cells. **(B)** Protein import rate constants (mean ± SEM) calculated from the accumulation curves. WT (k_I=2.08±0.08 ks^{-1}, n=50); (GS)$_4$-WT (k_I=2.52±0.08 ks^{-1}, n=125). Significance levels for two-tailed Mann-Whitney non-parametric test *P ≤0.05. P=7.00x10^{-3}. All points and bar plots indicate mean±SEM.

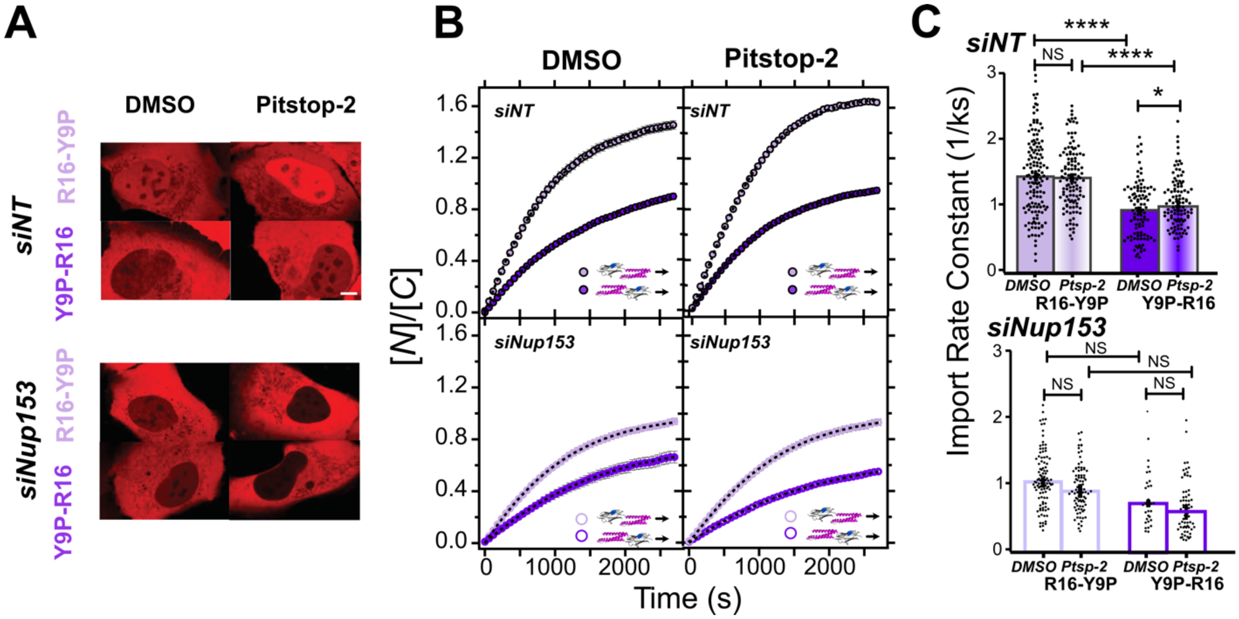

Supplementary figure 8. Exposure to Pitstop-2 maintains the NPC sensitivity to the mechano-directionality of the translocating cargo (A) Representative confocal images of U2OS cells 30 min in the recovery phase. Scale bar 10 µm. **(B)** Average time courses of the relative nucleus-to-cytoplasm localization of R16-Y9P and Y9P-R16 under normal conditions (siNT) and Nup153 knockdown (siNup153), and in the absence (DMSO) or presence (Pitstop-2). Pitstop-2 interferes with the NPC barrier, slightly increasing nuclear accumulation but showing no significant effect on the nuclear import kinetics. Knocking down Nup153 in cells exposed to Pitstop2 still abolishes the NPC sensitivity to the cargo's mechano-directionality, suggesting that Pitstop-2 does not interfere with Nup153. **(C)** Protein import rate constants (mean ± SEM) calculated from the accumulation curves. R16-Y9P (siNT; DMSO) (k_I=1.44±0.05 ks^{-1}, *n*=149); Y9P-R16 (siNT; DMSO) (k_I=0.92±0.04 ks^{-1}, *n*=101); R16-Y9P (siNT; Pitstop-2) (k_I=1.44±0.05 ks^{-1}, *n*=122); Y9P-R16 (siNT; Pitstop-2) (k_I=1.03±0.04 ks^{-1}, *n*=103); R16-Y9P (siNup153; DMSO) (k_I=1.10±0.05 ks^{-1}, *n*=105); Y9P-R16 (siNup153; DMSO) (k_I=0.77±0.08 ks^{-1}, *n*=31); R16-Y9P (siNup153; Pitstop-2) (k_I=0.96±0.05 ks^{-1}, *n*=35); Y9P-R16 (siNup153; Pitstop-2) (k_I=0.64±0.05 ks^{-1}, *n*=68); Significance levels for two-tailed Mann-Whitney non-parametric test NS> 0.05, **P* ≤0.05, *****P* ≤0.0001. siNT: DMSO R16-Y9P vs. Ptsp-2 R16-Y9P, *P*=0.98; DMSO Y9P-R16 vs. Ptsp-2 Y9P-R16, *P*=0.04; DMSO R16-Y9P vs. DMSO Y9P-R16, *P*=1.75x10^{-11}; Ptsp-2 R16-Y9P vs. Ptsp-2 Y9P-R16, *P*=2.40x10^{-10}. siNup153: DMSO R16-Y9P vs. Ptsp-2 R16-Y9P, *P*=0.06; DMSO Y9P-R16 vs. Ptsp-2 Y9P-R16, *P*=0.12; DMSO R16-Y9P vs. DMSO Y9P-R16, *P*=0.06; Ptsp-2 R16-Y9P vs. Ptsp-2 Y9P-R16, *P*=7.17x10^{-3}. All points and bar plots indicate mean±SEM.

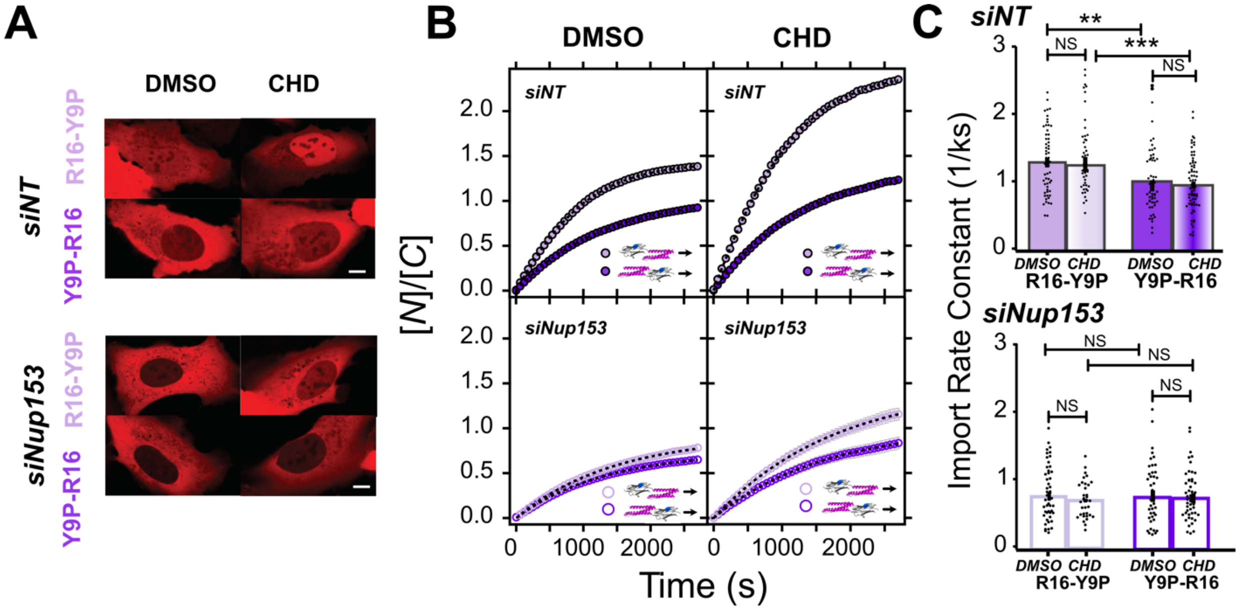

Supplementary figure 9. Exposure to trans-1,2-cyclohexanediol (CHD) maintains the NPC sensitivity to the mechano-directionality of the translocating cargo (A) Representative confocal images of U2OS cells 30 min in the recovery phase. Scale bar 10 µm. **(B)** Average time courses of the relative nucleus-to-cytoplasm localization of R16-Y9P and Y9P-R16 under normal conditions (siNT) and Nup153 knockdown (siNup153), and in the absence (DMSO) or presence (CHD). CHD interferes with the NPC barrier, greatly increasing nuclear accumulation but showing no significant effect on the nuclear import kinetics. Knocking down Nup153 in cells exposed to CHD still abolishes the NPC sensitivity to the cargo's mechano-directionality, suggesting that CHD does not interfere with Nup153. **(C)** Protein import rate constants (mean ± SEM) calculated from the accumulation curves. R16-Y9P (siNT; DMSO) (k_I=1.32±0.07 ks^{-1}, *n*=60); Y9P-R16 (siNT; DMSO) (k_I=1.02±0.06 ks^{-1}, *n*=58); R16-Y9P (siNT; CHD) (k_I=1.37±0.08 ks^{-1}, *n*=60); Y9P-R16 (siNT; CHD) (k_I=0.97±0.05 ks^{-1}, *n*=58); R16-Y9P (siNup153; DMSO) (k_I=0.84±0.06 ks^{-1}, *n*=51); Y9P-R16 (siNup153; DMSO) (k_I=0.85±0.06 ks^{-1}, *n*=43); R16-Y9P (siNup153; CHD) (k_I=0.70±0.04 ks^{-1}, *n*=35); Y9P-R16 (siNup153; CHD) (k_I=0.77±0.05 ks^{-1}, *n*=52); Significance levels for two-tailed Mann-Whitney non-parametric test NS> 0.05, ***P* ≤0.01, *** *P* ≤0.001. siNT: DMSO R16-Y9P vs. CHD R16-Y9P, *P*=0.77; DMSO Y9P-R16 vs. CHD Y9P-R16, *P*=0.82; DMSO R16-Y9P vs. DMSO Y9P-R16, *P*=9.64x10^{-4}; CHD R16-Y9P vs. CHD Y9P-R16, *P*=8.28x10^{-5}. siNup153: DMSO R16-Y9P vs. CHD R16-Y9P, *P*=0.23; DMSO Y9P-R16 vs. CHD Y9P-R16, *P*=0.23; DMSO R16-Y9P vs. DMSO Y9P-R16, *P*=0.80; CHD R16-Y9P vs. CHD Y9P-R16, *P*=0.45. All points and bar plots indicate mean±SEM.

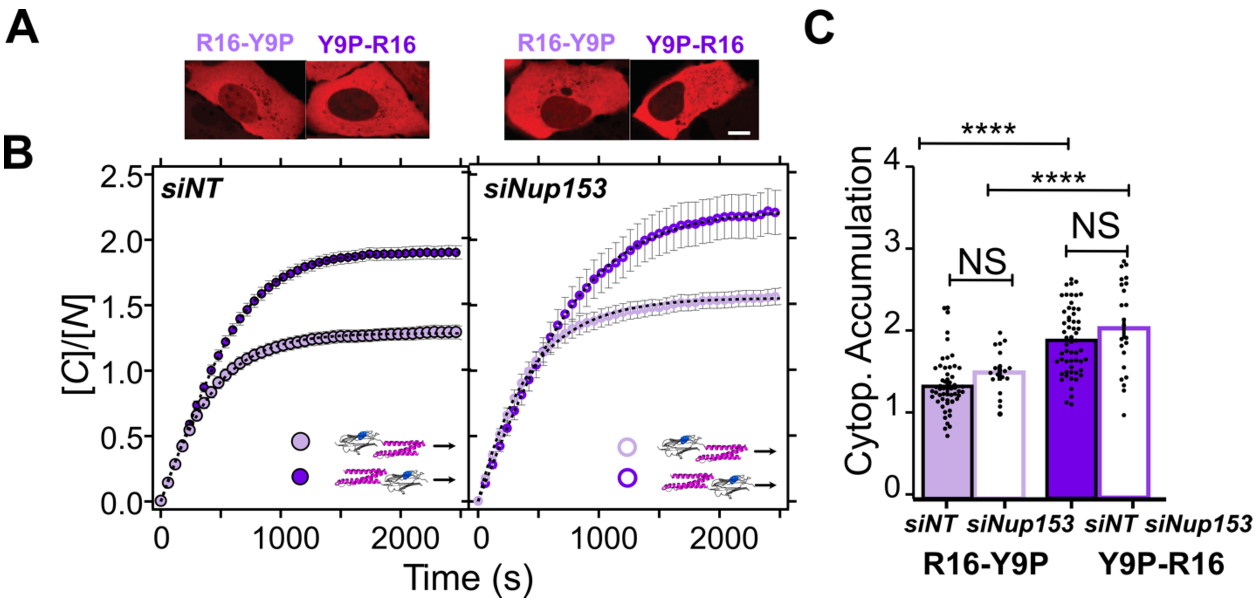

Supplementary figure 10. Silencing Nup153 does not abrogate the NPC sensitivity to the mechanical stability of the translocating cargos during export. (A) Representative confocal images of U2OS cells 30 min in the recovery phase. Scale bar 10 µm. **(B)** Average time courses of the relative cytoplasm-to-nucleus localization of R16-Y9P and Y9P-R16 under normal conditions (siNT) and Nup153 knockdown (siNup153). Note that, unlike the other time course curves, here cytoplasm-to-nucleus intensity is plotted. Nup153 does not significantly affect nuclear export, restricting the role of this Nup to nuclear import. **(C)** Cytoplasmic accumulation (mean ± SEM) calculated from the accumulation curves. R16-Y9P (siNT) (K_e=1.33±0.05, n=64); Y9P-R16 (siNT) (K_e=1.55±0.01, n=22); R16-Y9P (siNup153) (K_e=1.93±0.05, n=91); Y9P-R16 (siNup153) (K_e=2.06±0.16, n=27); Significance levels for Mann-Whitney non-parametric test. NS> 0.05, ****P ≤0.0001. siNT R16-Y9P vs. siNup153 R16-Y9P, P=0.06; siNT Y9P-R16 vs. siNup153 Y9P-R16, P=0.08; siNT R16-Y9P vs. siNT Y9P-R16, P=3.64x10^{-14}; siNup153 R16-Y9P vs. siNup153 Y9P-R16, P=5.57x10^{-5}. All points and bar plots indicate mean±SEM.

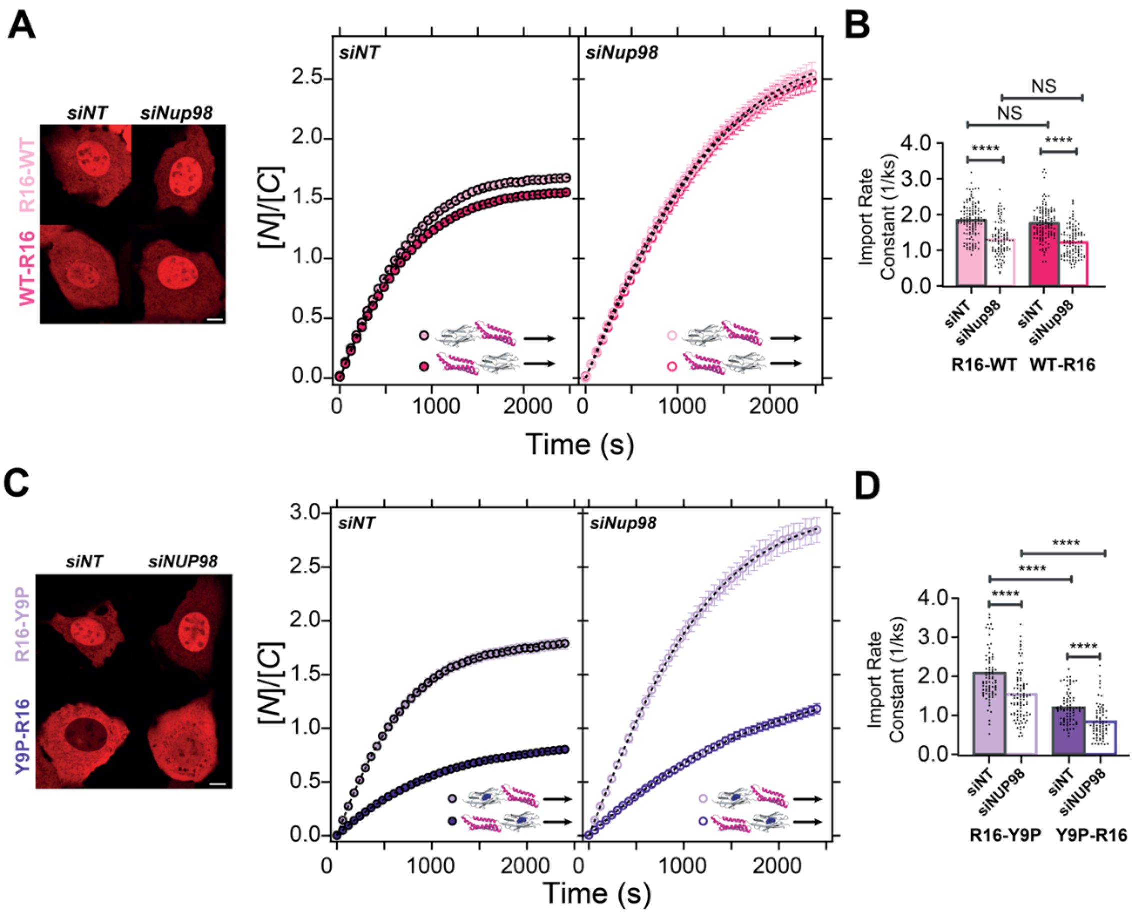

Supplementary figure 11. Silencing Nup98 decreases nuclear import kinetics and maintains the NPC sensitivity to the mechano-directionality of the translocating cargo. (A) Left: Representative confocal images of U2OS cells 30 min in the recovery phase. Scale bar 10 µm. Right: Average time courses of the relative nucleus-to-cytoplasm localization of R16-WT and WT-R16 under normal conditions (siNT) and Nup98 knockdown (siNup98). Knockdown of Nup98 decreases nuclear import kinetics, simultaneously increasing nuclear accumulation, but does not impact the NPC sensitivity to the mechano-directionality of the translocating cargo. **(B)** Protein import rate constants (mean ± SEM) calculated from the accumulation curves. R16-WT (siNT) (k_I=1.84±0.04 ks^{-1}, *n*=126); WT-R16 (siNT) *(k_I=1.75±0.05 ks^{-1}, n=127)*; R16-WT (siNup98) (k_I=1.30±0.05 ks^{-1}, *n*=96); WT-R16 (siNup98) (k_I=1.22±0.04 ks^{-1}, *n*=111). Significance levels for Mann-Whitney non-parametric test. NS> 0.05, *****P* ≤0.0001. siNT R16-WT vs. siNup98 R16-WT, *P*=1.15x10^{-13}; siNT WT-R16 vs. siNup98 WT-R16, *P*=8.54x10^{-28}; siNT R16-WT vs. siNT WT-R16, *P*=0.99; siNup98 R16-WT vs. siNup98, WT-R16, *P*=0.24. **(C)** Representative confocal images of U2OS cells 30 min in the recovery phase. Scale bar is 10 µm. Right: Average time courses of the relative nucleus-to-cytoplasm localization of R16-Y9P and Y9P-R16 under normal conditions (siNT) and Nup98 knockdown (siNup98). **(D)** Protein import rate constants (mean ± SEM) calculated from the accumulation curves. R16-Y9P (siNT) *(k_I=2.08±0.08 ks^{-1}, n=83)*; Y9P-R16 (siNT) (k_I=1.20±0.04 ks^{-1}, *n*=81); R16-Y9P (siNup98) (k_I=1.54±0.07 ks^{-1}, *n*=89); Y9P-R16 (siNup98) (k_I=0.83±0.05 ks^{-1}, *n*=75). Significance levels for Mann-Whitney non-parametric test. *****P* ≤0.0001. siNT R16-Y9P vs. siNup98 R16-Y9P, *P*=6.86x10^{-8}; siNT Y9P-R16 vs. siNup98 Y9P-R16, *P*=3.34x10^{-8}; siNT R16-Y9P vs. siNT Y9P-R16, *P*=8.88x10^{-16}; siNup98 R16-Y9P vs. Y9P-R16, *P*=4.76x10^{-13}. All points and bar plots indicate mean±SEM.

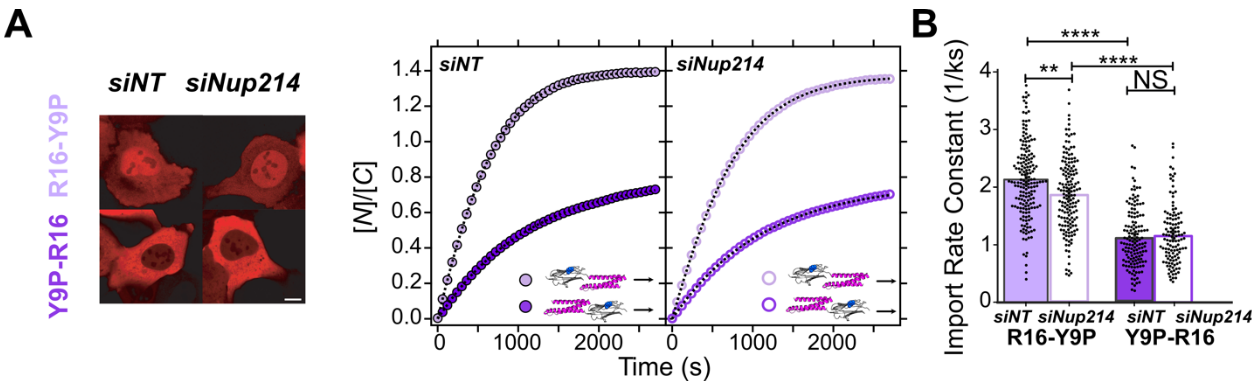

Supplementary figure 12. Silencing of Nup214 has just a minor effect on nuclear import kinetics and maintains the NPC sensitivity to the mechano-directionality of the translocating cargo. (A) Left: Representative confocal images of U2OS cells 30 min in the recovery phase. Scale bar 10 µm. Right: Average time courses of the relative nucleus-to-cytoplasm localization of R16-Y9P and Y9P-R16 under normal conditions (siNT) and Nup214 knockdown (siNup214). Depletion of Nup214 has a minor decreasing effect on the nuclear import kinetics, but maintains the NPC sensitivity to the mechano-directionality of the translocating protein cargo. **(B)** Protein import rate constants (mean ± SEM) calculated from the accumulation curves. R16-Y9P (siNT) (k_I=2.27±0.05 ks^{-1}, *n*=180); Y9P-R16 (siNT) (k_I=1.25±0.04 ks^{-1}, *n*=144); R16-Y9P (siNup214) (k_I=1.89±0.05 ks^{-1}, *n*=158); Y9P-R16 (siNup214) (k_I=1.17±0.05 ks^{-1}, *n*=119). Significance levels for two-tailedMann-Whitney non-parametric test NS> 0.05, **P≤0.01, ****P ≤0.0001. siNT R16-Y9P vs. siNup214 R16-Y9P, P=7.11x10^{-4}, siNT Y9P-R16 vs. siNup214 Y9P-R16, P=0.55; siNT R16-Y9P vs. siNT Y9P-R16 P=1.02x10^{-28}; siNup98 R16-Y9P vs. siNup98 Y9P-R16, P=1.11x10^{-15}. All points and bar plots indicate mean±SEM.

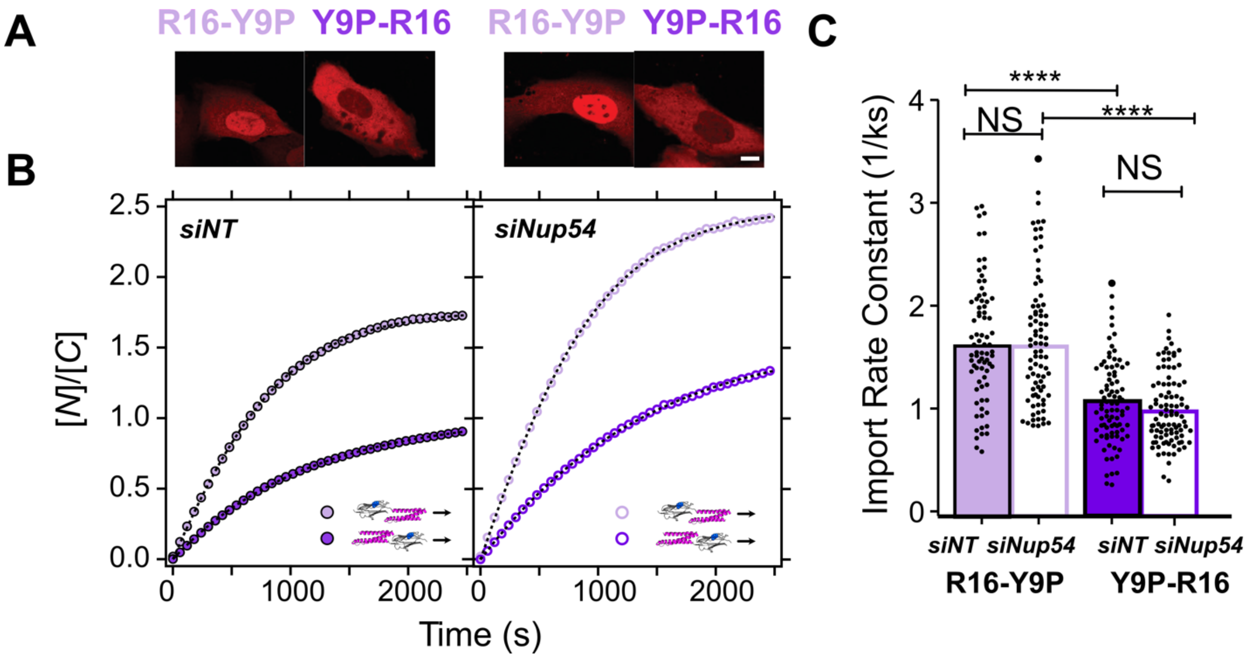

Supplementary figure 13. Silencing of Nup54 maintains the NPC sensitivity to the mechano-directionality of the translocating cargo. (A) Representative confocal images of U2OS cells 30 min in the recovery phase. Scale bar 10 µm. **(B)** Average time courses of the relative nucleus-to-cytoplasm localization of R16-Y9P and Y9P-R16 under normal conditions (siNT) and Nup54 knockdown (siNup54). Depletion of Nup54 has a minor decreasing effect on the nuclear import kinetics, but maintains the NPC sensitivity to the mechano-directionality of the translocating protein cargo. **(C)** Protein import rate constants (mean ± SEM) calculated from the accumulation curves. R16-Y9P (siNT) (k_I=1.65±0.07 ks^{-1}, *n*=77); Y9P-R16 (siNT) (k_I=1.06±0.04 ks^{-1}, *n*=87); R16-Y9P (siNup54) (k_I=1.64±0.09 ks^{-1}, *n*=87); Y9P-R16 (siNup54) (k_I=0.98±0.05 ks^{-1}, *n*=97). Significance levels for two-tailedMann-Whitney non-parametric test NS> 0.05, **P≤0.01, *****P* ≤0.0001. siNT R16-Y9P vs. siNup54 R16-Y9P, *P*=0.77, siNT Y9P-R16 vs. siNup54 Y9P-R16, *P*=0.16; siNT R16-Y9P vs. siNT Y9P-R16 *P*=4.71x10^{-11}; siNup54 R16-Y9P vs. siNup54 Y9P-R16, *P*=1.03x10^{-28}. All points and bar plots indicate mean±SEM.

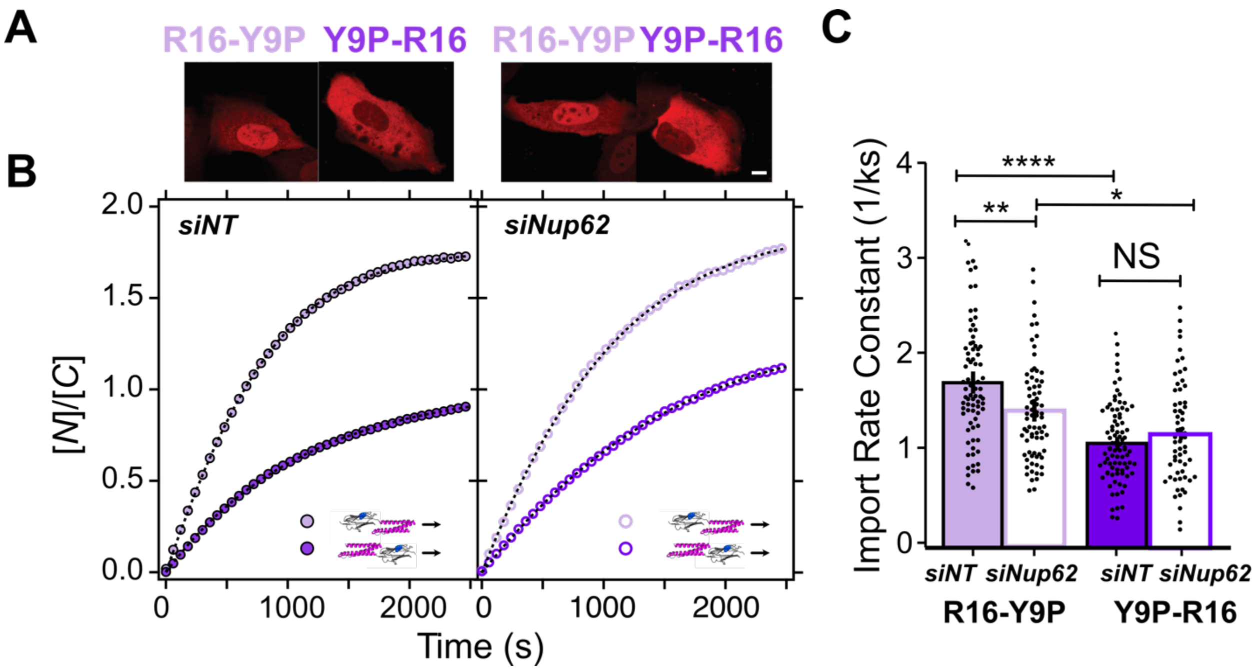

Supplementary figure 14. Silencing of Nup62 maintains the NPC sensitivity to the mechano-directionality of the translocating cargo. (A) Representative confocal images of U2OS cells 30 min in the recovery phase. Scale bar 10 µm. **(B)** Average time courses of the relative nucleus-to-cytoplasm localization of R16-Y9P and Y9P-R16 under normal conditions (siNT) and Nup62 knockdown (siNup62). Depletion of Nup62 has a minor decreasing effect on the nuclear import kinetics, but maintains the NPC sensitivity to the mechano-directionality of the translocating protein cargo. **(C)** Protein import rate constants (mean ± SEM) calculated from the accumulation curves. R16-Y9P (siNT) *(*k_I=1.65±0.07 ks^{-1}*, n*=77*)*; Y9P-R16 (siNT) *(*k_I=1.06±0.04 ks^{-1}*, n*=87*)*; R16-Y9P (siNup62) *(*k_I=1.32±0.06 ks^{-1}, *n*=78*)*; Y9P-R16 (siNup62) *(*k_I=1.15±0.05 ks^{-1}, *n*=64*)*. Significance levels for two-tailedMann-Whitney non-parametric test NS> 0.05, **P≤0.01, ****P ≤0.0001. siNT R16-Y9P vs. siNup65 R16-Y9P, P=2.34x10^{-4}, siNT Y9P-R16 vs. siNup62 Y9P-R16, P=0.07; siNT R16-Y9P vs. siNT Y9P-R16 P=4.71x10^{-11}; siNup62 R16-Y9P vs. siNup62 Y9P-R16, P=2.64x10^{-3}. All points and bar plots indicate mean±SEM.

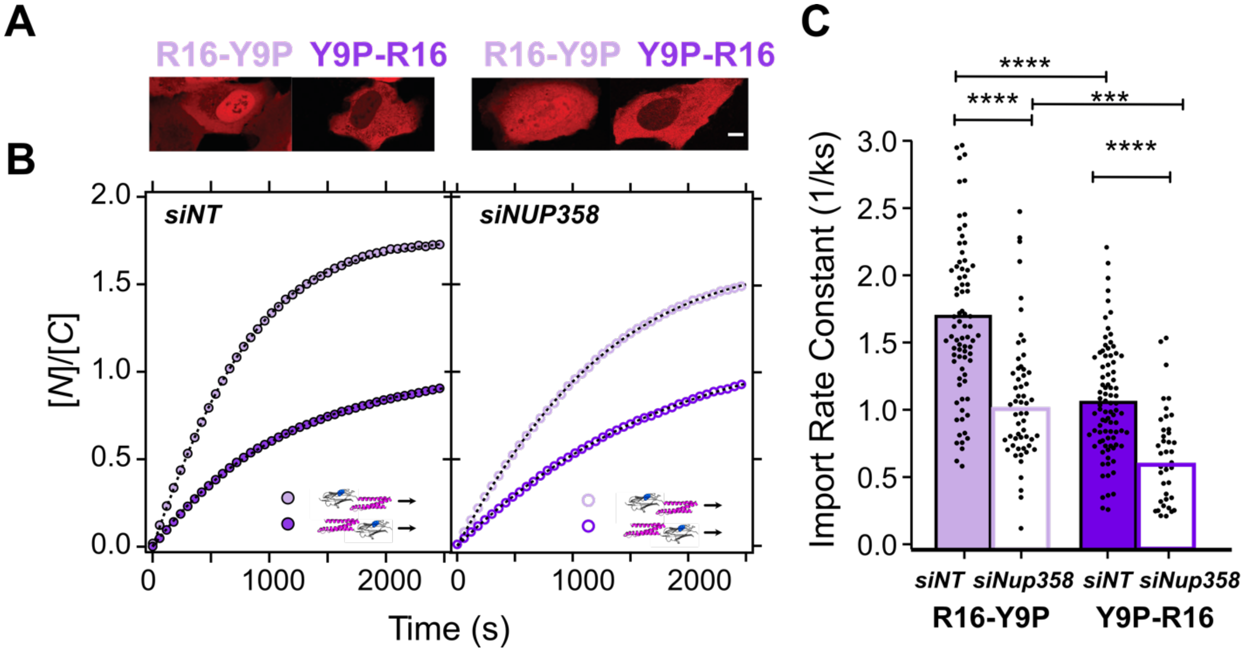

Supplementary figure 15. Silencing of Nup358 maintains the NPC sensitivity to the mechano-directionality of the translocating cargo. (A) Representative confocal images of U2OS cells 30 min in the recovery phase. Scale bar 10 µm. **(B)** Average time courses of the relative nucleus-to-cytoplasm localization of R16-Y9P and Y9P-R16 under normal conditions (siNT) and Nup358 knockdown (siNup358). Depletion of Nup358 has a minor decreasing effect on the nuclear import kinetics, but maintains the NPC sensitivity to the mechano-directionality of the translocating protein cargo. **(C)** Protein import rate constants (mean ± SEM) calculated from the accumulation curves. R16-Y9P (siNT) (k_I=1.65±0.07 ks^{-1}, *n*=77); Y9P-R16 (siNT) (k_I=1.06±0.04 ks^{-1}, *n*=87); R16-Y9P (siNup358) (k_I=1.09±0.08 ks^{-1}, *n*=56); Y9P-R16 (siNup358) (k_I=0.66±0.06 ks^{-1}, *n*=40). Significance levels for two-tailedMann-Whitney non-parametric test NS> 0.05, **P≤0.01, ****P ≤0.0001. siNT R16-Y9P vs. siNup358 R16-Y9P, P=1.34x10^{-8}, siNT Y9P-R16 vs. siNup358 Y9P-R16, P=6.53x10^{-7}; siNT R16-Y9P vs. siNT Y9P-R16 P=4.71x10^{-11}; siNup358 R16-Y9P vs. siNup358 Y9P-R16, P=8.68x10^{-6}. All points and bar plots indicate mean±SEM.

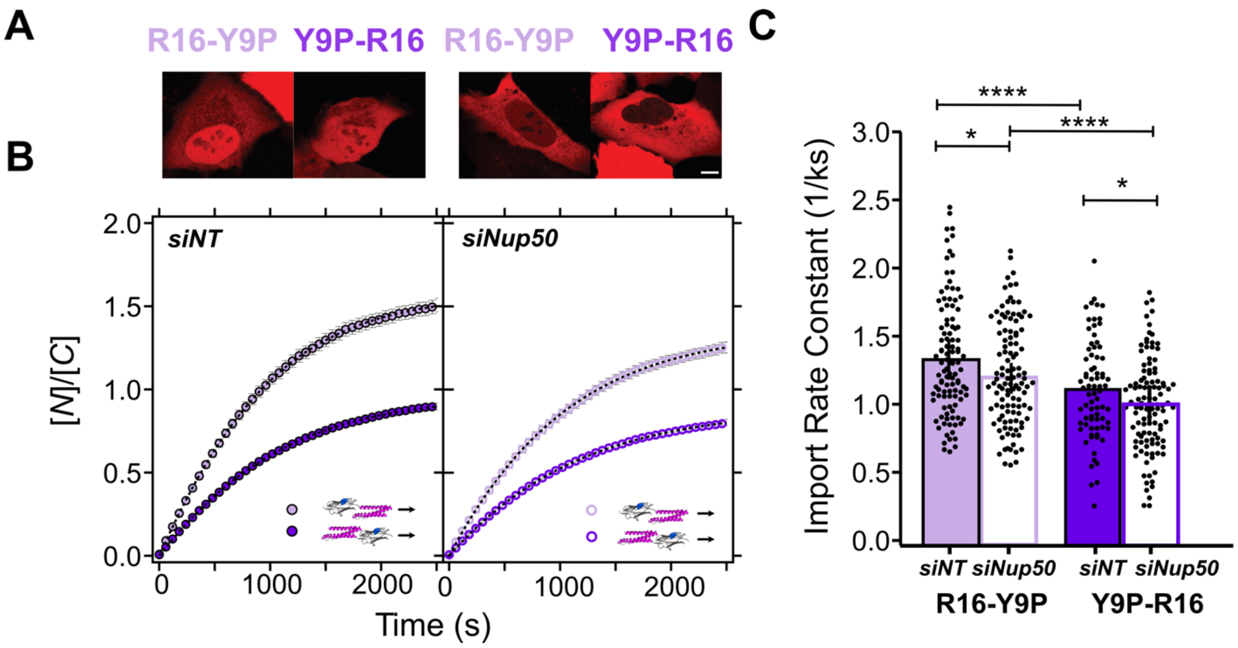

Supplementary figure 16. Silencing of Nup50 maintains the NPC sensitivity to the mechano-directionality of the translocating cargo. (A) Representative confocal images of U2OS cells 30 min in the recovery phase. Scale bar 10 µm. **(B)** Average time courses of the relative nucleus-to-cytoplasm localization of R16-Y9P and Y9P-R16 under normal conditions (siNT) and Nup50 knockdown (siNup50). Depletion of Nup50 has a minor decreasing effect on the nuclear import kinetics but maintains the NPC sensitivity to the mechano-directionality of the translocating protein cargo. **(C)** Protein import rate constants (mean ± SEM) calculated from the accumulation curves. R16-Y9P (siNT) (k_I=1.40±0.07 ks^{-1}, *n*=111); Y9P-R16 (siNT) (k_I=1.12±0.04 ks^{-1}, *n*=77); R16-Y9P (siNup50) (k_I=1.22±0.05 ks^{-1}, *n*=115); Y9P-R16 (siNup50) (k_I=0.98±0.03 ks^{-1}, *n*=112). Significance levels for two-tailedMann-Whitney non-parametric test NS> 0.05, **P≤0.01, ****P ≤0.0001. siNT R16-Y9P vs. siNup50 R16-Y9P, P=0.02, siNT Y9P-R16 vs. siNup50 Y9P-R16, P=0.02; siNT R16-Y9P vs. siNT Y9P-R16 P=4.84x10^{-5}; siNup50 R16-Y9P vs. siNup50 Y9P-R16, P=3.50x10^{-5}. All points and bar plots indicate mean±SEM.

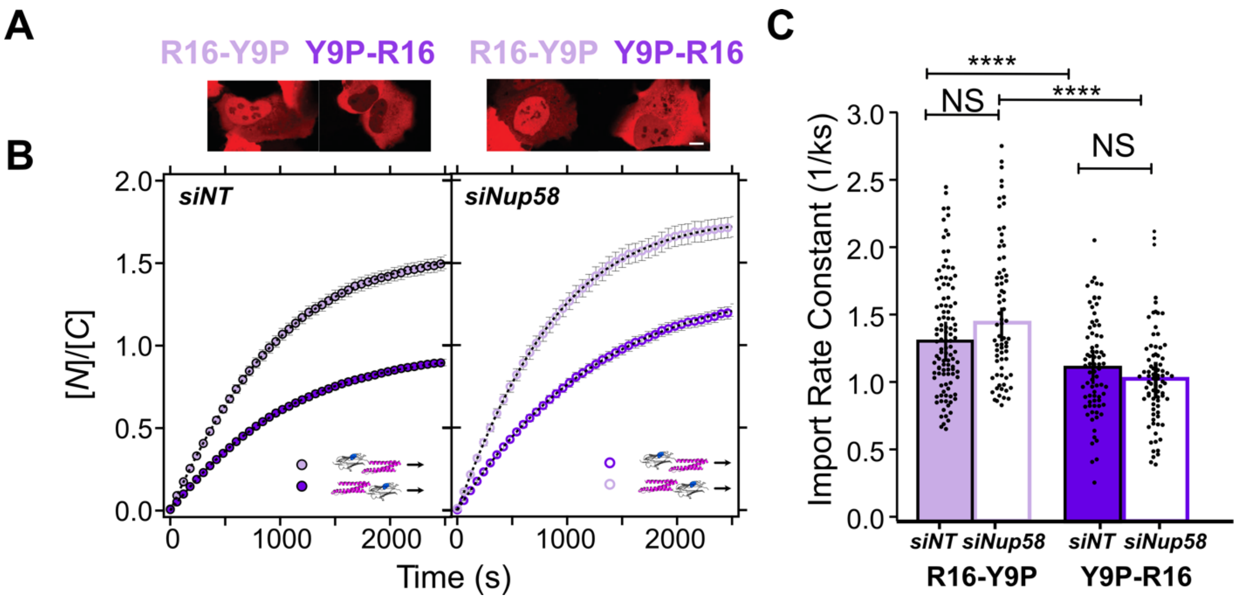

Supplementary figure 17. Silencing of Nup58 maintains the NPC sensitivity to the mechano-directionality of the translocating cargo. (A) Representative confocal images of U2OS cells 30 min in the recovery phase. Scale bar 10 µm. **(B)** Average time courses of the relative nucleus-to-cytoplasm localization of R16-Y9P and Y9P-R16 under normal conditions (siNT) and Nup58 knockdown (siNup58). Depletion of Nup58 has a minor decreasing effect on the nuclear import kinetics, but maintains the NPC sensitivity to the mechano-directionality of the translocating protein cargo. **(C)** Protein import rate constants (mean ± SEM) calculated from the accumulation curves. R16-Y9P (siNT) *(*k_I=1.40±0.06 ks^{-1}, *n*=110*)*; Y9P-R16 (siNT) *(*k_I=1.11±0.04 ks^{-1}*, n*=75*)*; R16-Y9P (siNup58) *(*k_I=1.52±0.06 ks^{-1}, *n*=70*)*; Y9P-R16 (siNup58) *(*k_I=1.06±0.05 ks^{-1}, *n*=80*)*. Significance levels for two-tailedMann-Whitney non-parametric test NS> 0.05, **P≤0.01, ****P ≤0.0001. siNT R16-Y9P vs. siNup58 R16-Y9P, *P*=0.07, siNT Y9P-R16 vs. siNup58 Y9P-R16, *P*=0.18; siNT R16-Y9P vs. siNT Y9P-R16 *P*=4.84x10^{-5}; siNup58 R16-Y9P vs. siNup58 Y9P-R16, *P*=1.24x10^{-8}. All points and bar plots indicate mean±SEM.

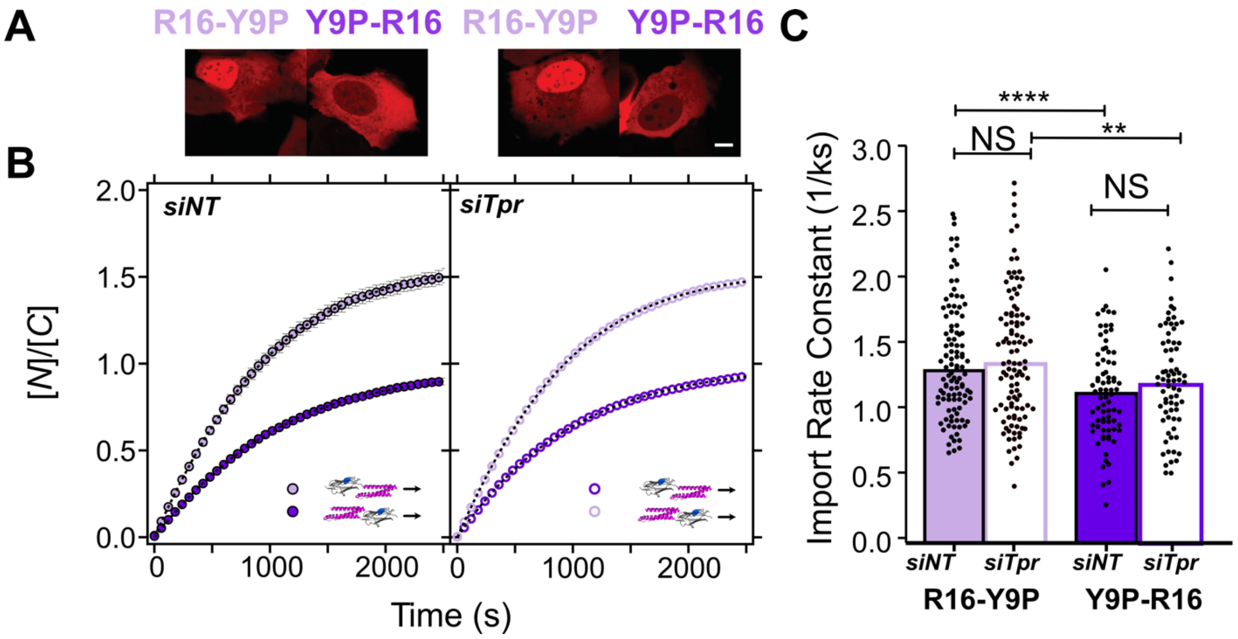

Supplementary figure 18. Silencing of Tpr maintains the NPC sensitivity to the mechano-directionality of the translocating cargo. (A) Representative confocal images of U2OS cells 30 min in the recovery phase. Scale bar 10 µm. **(B)** Average time courses of the relative nucleus-to-cytoplasm localization of R16-Y9P and Y9P-R16 under normal conditions (siNT) and Tpr knockdown (siTpr). Depletion of Tpr has a minor decreasing effect on the nuclear import kinetics but maintains the NPC sensitivity to the mechano-directionality of the translocating protein cargo. **(C)** Protein import rate constants (mean ± SEM) calculated from the accumulation curves. R16-Y9P (siNT) *(*k_I=1.40±0.07 ks^{-1}*, n*=111*)*; Y9P-R16 (siNT) *(*k_I=1.12±0.04 ks^{-1}*, n*=77*)*; R16-Y9P (siNup358) *(*k_I=1.44±0.05 ks^{-1}, *n*=111*)*; Y9P-R16 (siTpr) *(*k_I=1.15±0.04 ks^{-1}, *n*=74*)*. Significance levels for two-tailedMann-Whitney non-parametric test NS> 0.05, **P≤0.01, ****P ≤0.0001. siNT R16-Y9P vs. siTpr R16-Y9P, P=0.29, siNT Y9P-R16 vs. siNupTpr Y9P-R16, P=0.35; siNT R16-Y9P vs. siNT Y9P-R16 P=4.84x10^{-5}; siTpr R16-Y9P vs. siNup58 Y9P-R16, P=3.59x10^{-3}. All points and bar plots indicate mean±SEM.

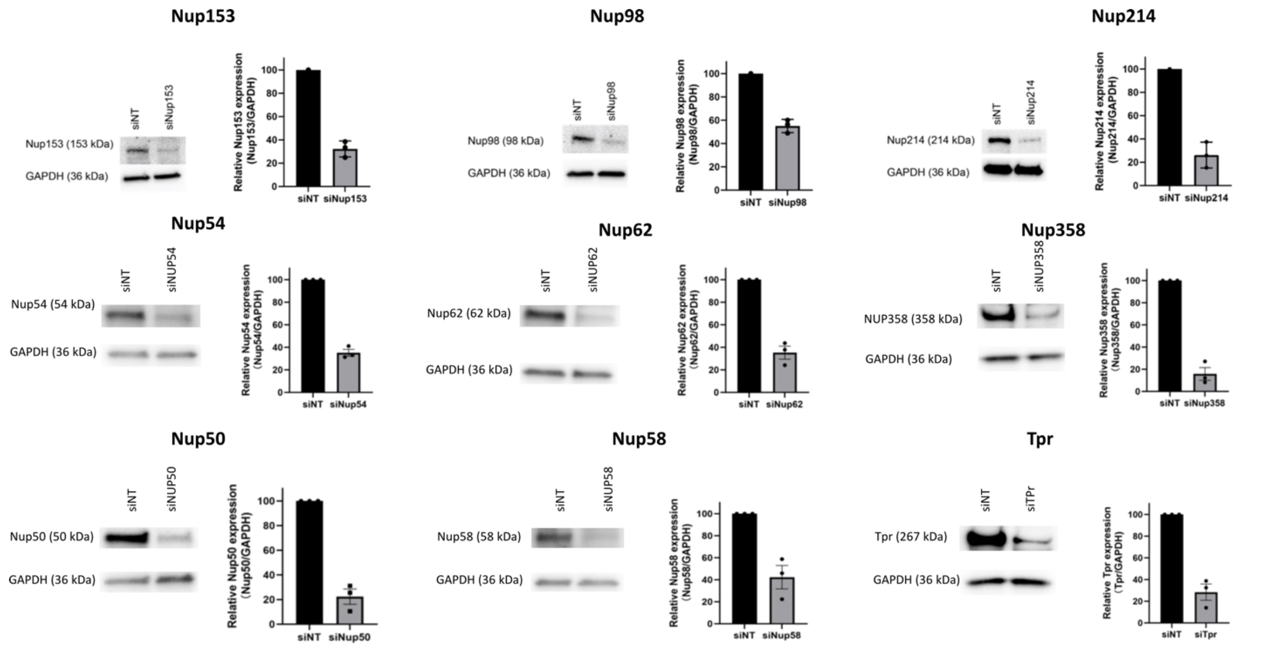

Supplementary figure 19. Expression of Nup153, Nup98, Nup214, Nup54, Nup62, Nup358, Nup50, Nup58, and Tpr in U2OS in Nup-silencing experiments. Each panel: (left) representative immunoblotting analysis and (right) relative expression of the corresponding FG-Nup normalized by GAPDH. siNT: non-targeting siRNA. siNup: nucleoporin-targeting siRNA. The datapoints represent independent experiments. Error bars are SD.

Supplementary Table S1. Experimental acquisition protocols used for LEXY experiments. The activation and recovery phases of the protocols were adjusted to the number of domains added to the LEXY vector. The activation phase is conducted under continuous blue light illumination.

	**Acquisition length**	**Acquisition frequency**
**Activation period**		
1-8 domains	10 min	frame / min
**Recovery period**		
1-3 domains	45 min	frame / min
4 domains	56 min	frame / min
5 domains	72 min	frame / 1.5 min
6 domains	92 min	frame / 2.5 min
7 domains	120 min	frame / 3 min
8 domains	152 min	frame / 3.5 min

Supplementary Table S2. Primer sequences used for RT-qPCR experiments.

Primer name	Sequence (5’-3’)
SRF-F	TCACCTACCAGGTGTCGGAGTC
SRF-R	GTGCTGTTTGGATGGTGGAGGT
MYL9-F	GGATGTGATTCGCAACGCCTTTG
MYL9-R	CGGTACATCTCGTCCACTTCCT
GAPDH-F	GTCTCCTCTGACTTCAACAGCG
GAPDH-R	ACCACCCTGTTGCTGTAGCCAA
